# An apparent-time study of an ongoing sound change in Seoul Korean: A prosodic account

**DOI:** 10.1371/journal.pone.0240682

**Published:** 2020-10-22

**Authors:** Jiyoun Choi, Sahyang Kim, Taehong Cho

**Affiliations:** 1 Department of Social Psychology, Sookmyung Women’s University, Seoul, Korea; 2 Department of English Education, Hongik University, Seoul, Korea; 3 Department of English Language and Literature, Hanyang Institute for Phonetics and Cognitive Science, Hanyang University, Seoul, Korea; Universite Sorbonne Nouvelle Paris 3, FRANCE

## Abstract

In present-day Seoul Korean, the primary phonetic feature for the lenis–aspirated stop distinction is shifting from VOT to F0. Some previous studies have considered this sound change to be a tonogenesis, whereby the low-level F0 perturbation has developed into tonal features (L for the lenis and H for the aspirated) in the segmental phonology. They, however, have examined the stop distinction only at a phrase- or utterance-initial position. We newly explore the sound change in relation to various prosodic structural factors (position and prominence). Apparent-time production data were recorded from four speaker groups: young female, young male, old female, old male. The way the speakers use VOT versus F0 indeed varies as a function of position and prominence. Crucially, in all groups, VOT is still used for the lenis–aspirated distinction phrase-medially due to the lenis stop voicing. This role of VOT, however, is found only in the non-prominent (unfocused) condition, in which the F0 difference is reduced to a low-level perturbation effect. In the prominent (focused) context in which tones come into play, the role of VOT diminishes, led by young female speakers. These can be interpreted as a prosodically-conditioned, complementary use of the features to maintain sufficient contrast. Importantly, however, the tonal difference under focus is not bidirectionally polarized, so that F0 is not lowered for the lenis stop. A lack of direct enhancement of the distinctive L tone weakens a possibility that F0 is transphonologized to the phonemic feature system of the language. As an alternative to the view that tonal features are newly introduced in the segmental phonology, we propose a prosodic account: the sound change is best characterized as a prosodically-conditioned change in the use of the segmental voicing feature (implemented by VOT) versus already available post-lexical tones in the intonational phonology of Korean.

## 1. Introduction

Present-day Seoul Korean is undergoing a sound change in the use of the phonetic features that distinguish the lenis stop from the aspirated stop. Until the early 2000s, researchers reported that the stops could be distinguished at least statistically by the segmental voicing feature of the voice onset time (VOT), with the longest VOT used for the aspirated stop, the intermediate for the lenis stop, and the shortest for the fortis stop (see e.g. [1], and references therein). Along with the segmental voicing feature (i.e., the VOT), the fundamental frequency (F0) of the following vowel has been regarded as a secondary phonetic feature attributable to the low-level phonetic process, so that the aspirated and the fortis stops are produced with high F0, reflecting laryngeal tension, whereas the lenis stop has been produced with low F0, reflecting laryngeal laxness. However, a growing body of phonetic research has since evidenced the possible beginning of a sound change reversing the primacy of the two phonetic features VOT and F0 in phonologically distinguishing the lenis and aspirated stops. Young, especially female, speakers of Seoul Korean use F0 systematically as the primary phonetic feature for the lenis–aspirated distinction, whereas the use of VOT has been minimized in or has disappeared from their production (e.g., [2–6]). In the vanguard of exploring the sound change [2], noted that young speakers used the F0 difference more extensively, driven by the VOT merger in progress: these speakers’ VOTs for the lenis stop had lengthened to the extent that they completely overlapped the VOTs of the aspirated stop. Subsequently, some researchers interpreted the ‘emergence’ of F0 in place of VOT as indicating that a ‘tonogenetic’ sound change is underway in Seoul Korean (e.g., [5, 6]). The tonogenetic account proposed regarding the noted change is that the phonetically driven low-level F0 differences (due to differential laryngeal tension) have been exaggerated and transferred to phonological contrasts, introducing bifurcated tonal features (H vs. L) in the segmental phonology of Seoul Korean, while the role of the segmental voicing feature (as reflected in VOT) is diminishing and eventually leading to a VOT merger (see [7] for a review of the typology of tonogenesis; and [8] for a similar case in Afrikaans). This kind of change in the cue primacy (from VOT to F0) at the level of segmental phonology is referred to as transphonologization [6]. Writes “[t]he change in Seoul Korean essentially exhibits the same type of transphonologization we find in cases of ‘tonogenesis’ reported in the literature (e.g. Khmer, Afrikaans), where f0 shifts from a redundant phonetic property of a laryngeal contrast to a primary cue” (p.121).

Although the aforementioned studies have provided valuable linguistic insights into the putatively tonogenetic sound change that may be underway in Seoul Korean [2], deferred making any claim that the process be tonogenesis given the existence of other phonetic correlates of the stop contrasts arising from consonantal phonation differences. Moreover, the scope of the existing studies has been limited to the stops produced in a particular prosodic context, namely the utterance-initial or phrase-initial position. We are therefore left with many important questions open as to the exact nature of the sound change in progress, especially in relation to prosodic structure. Understanding the ongoing sound change in various prosodic structural contexts is particularly important given the literature on the phonetics–prosody interface, which suggests that the phonetic realization of phonological contrast is modulated systematically by prosodic structure in which contrasting sounds occur ([9–12]). The crux of the phonetics–prosody interface is that prosody is a grammatical entity in its own right (e.g., [13]) and serves as an integral part of speech production and perception, so that no sound pattern can be understood in full without making reference to the phonetics–prosody interface (e.g., [14–17], *inter alia*). An important question is then how the differential use of the phonetic features VOT and F0 for the lenis–aspirated distinction in Seoul Korean can be understood in terms of prosodic–structural conditioning of their uses. The purpose of the present study is to answer this fundamental question and to illuminate the exact nature of the purported tonogenetic sound change in Seoul Korean in a manner that includes consideration of the phonetics–prosody interface. To this end, the present study will examine how the two phonetic features VOT and F0 are employed to maintain the lenis–aspirated distinction in different prosodic structures in which the stops occur; and how the use of VOT and F0 in relation to prosodic structure may differ among speakers according to their age and gender.

An important aspect of the phonetics–prosody interface is a position-related domain-initial strengthening phenomenon—i.e., the realization of the phonetic feature is ‘strengthened’ at the beginning of a larger prosodic constituent (e.g., an Intonational Phrase) relative to a smaller one (a Prosodic Word embedded in a phrase) (e.g., [9, 18–21]). An earlier study by [9], for example, demonstrated that VOTs for both the lenis and the aspirated stops are longer phrase-initially than phrase-medially, and that a VOT difference is maintained between the two categories across different prosodic positions. Furthermore, the VOT difference used to maintain the lenis–aspirated distinction is likely to be larger phrase-medially, especially within an Accentual Phrase (AP; larger than a prosodic word but smaller than an Intonational Phrase in Seoul Korean). This is because the lenis stop undergoes a further weakening process known as the lenis stop voicing rule, by which it becomes voiced intervocalically (e.g., [12, 20]), leading to maintenance of sufficient phonetic contrast between the lenis and aspirated stops along the phonetic continuum of the segmental voicing feature of VOT. It is therefore reasonable to assume that the role of VOT in the lenis–aspirated distinction may not have been diminishing in its entirety but may instead differ as a function of prosodic position, necessitating further research on the ongoing sound change to include consideration of the interaction between segmental phonology and prosodic structure.

Another important aspect of the phonetics–prosody interface especially in relation to Seoul Korean is that the use of F0 for the lenis–aspirated distinction that is originally driven by the low-level phonetic process is closely related to the post-lexical intonational phonology of Seoul Korean. The intonational structure of an AP is generally assumed to be specified with TH…LH, where ‘T’ is assigned an H tone if the initial segment is an aspirated or a fortis consonant and assigned an L tone elsewhere including when it is a lenis stop (e.g., [12, 22, 23]). This means that the L and H tones associated with the phrase-initial lenis and aspirated stops are not attributable to the segmental phonology, but are assigned post-lexically to syllables in an AP-initial (or IP-initial position) by the intonational phonology of the language. This can be interpreted as illustrating another type of tonogenesis at the level of intonational phonology, in the sense that F0 perturbation due to a low-level phonetic effect has developed into tonal contrasts being phonologized in the intonational phonology of a language [24]. In contrast, the phrase-medial use of F0 for the lenis–aspirated distinction is not tightly linked to the intonational phonology since the distribution of phrase-medial (non-edge) tones (as marked by “T**H**…**L**H”) is opaque to the segmental phonology, showing no post-lexical segment–tone interaction. This aspect of the phonetics–prosody interface therefore necessitates an exploration of the ongoing sound change in terms of how the segment–tone interaction is conditioned by prosodic structure in relation to the intonational phonology of Seoul Korean, and how such interaction conditions the ongoing sound change.

With all these aspects of the phonetics–prosody interface taken into account with regard to Seoul Korean, it is reasonable to assume that the use of the segmental voicing feature (VOT) and the tonal feature (F0) for the lenis–aspirated distinction varies depending on the prosodic positions in which they occur, and that the ongoing sound change that has been led by young female speakers of Seoul Korean has its underpinnings stemming from the specificity of the phonetics–prosody interface in Seoul Korean. More specifically, one might hypothesize that the use of the F0 feature for the stop contrast may diminish in the phrase-medial position as compared to that in the phrase-initial position, not only because the voicing difference is maintained phrase-medially due to lenis stop voicing (thus the F0 difference becomes redundant), but also because the use of F0 for the phrase-medial stop contrast may be overridden by the intonational phonology (as there is no post-lexical segment–tone interaction in phrase-medial positions). If this is the case, one might further argue that the change in the primacy of the phonetic features is better characterized as a consequence of differential use of the segmental voice feature (e.g., VOT) versus the post-lexically assigned tonal (F0) feature as a function of prosodic structure, which may also differ across age and gender groups, giving rise to the sort of ongoing sound change under investigation here. The present study is one of the first apparent-time studies, if not the first, that explore these possibilities. That is, it investigates how the ongoing sound change in present-day Seoul Korean may be understood in relation to the phonetics–prosody interface by examining the use of the segmental voice feature (VOT) and the tonal (F0) feature in phrase-initial and phrase-medial positions, and how the prosodic-structurally conditioned use of the two phonetic features may differ across gender and age groups.

Finally, the present study also explores the differential use of the segmental voicing feature of VOT and the tonal feature in relation to prominence. Although Korean is often classified as an edge-prominence language in which prominence is expressed primarily by the phrasal edge tones [25], the prominence may also come from information structure due to narrow or contrastive focus on a particular syllable or a word (e.g., [20]). Focus-induced prominence generally entails strengthening of language-specific phonetic features, which often tends to enhance phonemic contrasts in a given language (e.g., [10, 11, 26–30]). For example, de Jong and his colleague [11, 27] demonstrated that although both English and Arabic show coda voicing effects on the preceding vowel duration (longer vowels before voiced consonants), the durational difference, which may have originated from a low-level phonetic process, is enhanced under focus in English but not in Arabic, in which the duration is reserved for making phonological quantity contrast. They suggested that the focus-related prominence induces “localized hyperarticulation” of a particular segment or syllable, which can be used as a diagnostic for the phonological use of the phonetic content (or the phonetic feature) [31]. Also examined the prominence effect on the phonetic realization of tonal (F0) features in Mandarin Chinese, and showed that the prominence led to a bidirectional enhancement of F0 values in opposite directions—i.e., with F0 being higher for rising tones but lower for falling tones under focus. More recently [29], reported similar effects in Kyungsang (“Gyeongsang”) Korean (a lexical pitch accent variety of Korean, similar to Japanese), showing a bidirectional polarization of the tonal contrast in which an H tone and an L tone of lexical pitch accents are produced with even higher F0 and even lower F0, respectively, in the focused than in the unfocused condition. The authors interpreted this bidirectional polarization of F0 contrast as suggesting that the targets of lexically specified tones (H or L) are phonologically enhanced in the prominent (focused) condition, which ultimately maximizes lexical distinction.

In a similar vein, investigating the effect of focus on the use of F0 in Seoul Korean will allow us to evaluate the phonological status of the F0 feature in association with the purportedly tonogenetic sound change. If the tonal features have been introduced into the feature system of the segmental phonology in Seoul Korean, we might observe a similar bidirectional enhancement with a polarization of F0 in the prominent (focused) condition—i.e., with F0 being higher for the aspirated but lower for the lenis stop regardless of whether the stops occur phrase-initial or phrase-medially. On the other hand, if tones are purely post-lexically assigned by the intonational phonology as discussed earlier, and therefore have not been internalized in the distinctive feature system of the segmental phonology, there is no a priori reason to expect bidirectional F0 enhancement.

Other languages that employ post-lexically specified tonal contrasts (e.g., English and German; [32, 33]) in fact show a tendency that F0 for both L and H tones (or in falling and rising tones) increases in the prominent context, and the magnitude of F0 increase is larger for the H or rising tone, resulting in a unidirectional enhancement of the post-lexical tonal contrast. Similarly, it is predicted that the F0 associated with post-lexical tones, if assigned by the intonational phonology of Seoul Korean, will also increase under focus for both the L and H tones, though the F0 difference between them may still increase in the focused condition.

In sum, the present study documents the apparent-time variation in use of the segmental voicing feature (VOT) and the F0 feature by comparing production data obtained from four speaker groups in present-day Seoul Korean (young female, young male, older female, and older male speakers), and explore the questions that have been discussed. The apparent-time production data will allow us to explore how the assumed differential use of the segmental voice feature (VOT) versus the tonal feature (F0) for the lenis–aspirated contrast in relation to prosodic structure (position and prominence) may differ as a function of age and gender. The data may therefore meaningfully map out the ongoing sound change with consideration given to the phonetics–prosody interface, illuminating how the incipient sound change led by innovative young female speakers may transmit to other groups in the speech community, particularly in terms of how the differential use of the available phonetic cues for the lenis–aspirated distinction as a function of prosodic structure may change over time across generations.

## 2. Methods

### 2.1. Participants and recording

A total of 32 native speakers of Seoul Korean (16 females and 16 males) participated in an acoustic recording session conducted in 2017. They were born and raised in Seoul or in Gyeonggi Province (the area surrounding Seoul). Half of the participants (8 females and 8 males) were born between 1992 and 1998 (mean age of 22), forming the young female and male speaker groups. The other half (8 females and 8 males) were born between 1954 and 1961 (mean age of 59), forming the old female and male speaker groups. The participants were not aware of the purpose of the study and were paid for their participation. None of the participants reported any hearing or speech problem. The acoustic data were recorded at a sampling rate of 44 kHz, using a Tascam Hd-P2 digital recorder and a SHURE KSN44 microphone, in a sound-attenuated booth at the Hanyang Institute for Phonetics and Cognitive Science of Language in Seoul. This study was reviewed and approved by the committee of the internal review board of HICPS (Hanyang Institute for Phonetics & Cognitive Sciences of Language). The participants have signed a consent form to participate in the research; and the acoustic data were analyzed anonymously.

### 2.2. Speech materials and procedure

**Fig 1:**
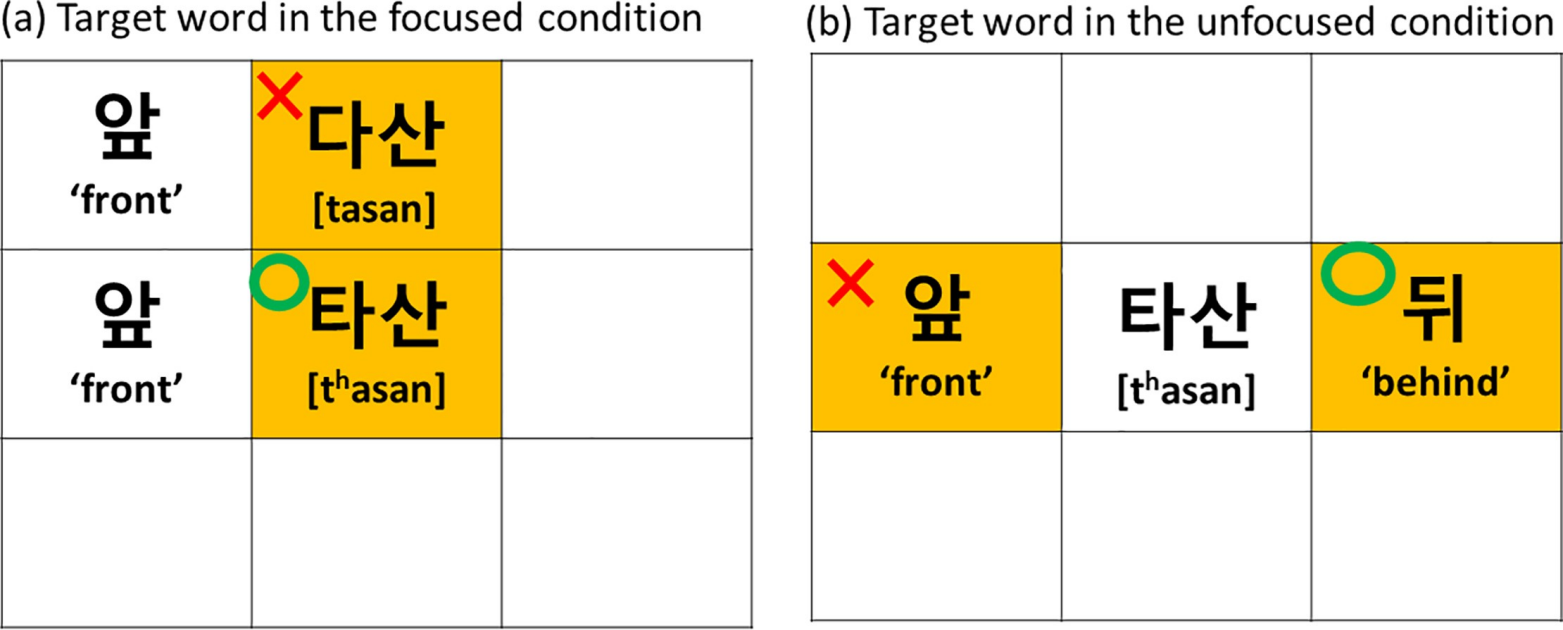
An illustration of the board game situations used to create a mini dialogue context in which the target word (*t*^*h*^*asan*) would receive (a) a contrastive (corrective) focus and (b) no focus; in the latter case the focus would fall on the following word ‘behind’. See the text for detail on the elicitation procedure.

The target stops were controlled to be the alveolar stops (aspirated /t^h^/ and lenis /t/) in the /a/ context (/t^h^a, ta/). A total of eight minimal pairs of disyllabic words were prepared having contrastive stops in the word-initial positions, as listed in Table 1. The test words were embedded in carrier sentences in a mini discourse situation in which the boundary conditions (IP-initial vs. IP-medial) and the focus conditions (focus vs. no focus) could be manipulated. Table 2 gives an example set of sentences bearing a test word /t^h^asan/. The mini discourse situation guided the speaker to produce an answer to a question with an intended prosodic condition (2 boundary conditions × 2 focus conditions). To induce the intended rendition as naturally as possible, we created a board game situation in which the speaker was instructed to answer a question by indicating where to put a word card on the board. As shown in Fig 1, the speaker was first shown the board on a computer screen, which indicated the correct location where the next card should be placed. (Note that on the board the ‘red’ cross indicates a wrong location and the green circle a correct location.) The speaker was then presented with a pre-recorded question of whether the interlocutor (the questioner) should put the card at a particular (wrong) location as, for example, written in Table 2A, “*Shall I put this card (or the card to be picked up this time) in front of ‘tasan’*?*”*. Since the correct location (marked by a green circle) shown on the board was not in front of ‘tasan’ but of ‘t^h^asan’ (Fig 1A), the speaker would correct the interlocutor by saying *“No*, *put it in front of ‘t*^*h*^*asan’ this time”* (as written in Table 2A). In this way, the target word ‘t^h^asan’ received (corrective) contrastive focus (the focus condition). In responding to a similar question, if the information provided on the board indicated that the correct location was not in front of the target word but behind it, the speaker corrected the interlocutor by saying, *“No*, *put it behind ‘t*^*h*^*asan’ this time”* (as written in Table 2B). In this case, the contrastive focus fell on “behind”, so that the target word was unfocused (the no-focus condition). Note that, to induce more natural and more spontaneous speech than that induced by a simple reading task, neither the prompt (question) sentence nor the target-bearing carrier sentence was written in full on the screen, and the speaker relied on the auditory prompt and the information provided on the computer screen.

**Table 1:** Minimal pairs for the word-initial lenis–aspirated contrast.

Lenis	Aspirated	Lenis	Aspirated
/tanto/ ‘short sword’	/t^h^anto/ ‘trajectory’	/tato/ ‘tea ceremony’	/t^h^ato/ ‘overthrow’
/talak/ ‘attic’	/t^h^alak/ ‘corruption’	/tasan/ ‘fecundity’	/t^h^asan/ ‘calculation’
/tawʌn/ ‘pluralism’	/t^h^awʌn/ ‘ellipse’	/tanso/ ‘bamboo flute’	/t^h^anso/ ‘carbon’
/tansik/ ‘fast’	/t^h^ansik/ ‘sigh’	/tatʃak/ ‘prolificacy’	/t^h^atʃak/ ‘threshing’

**Table 2:** Example sentences with the test word /*t*^*h*^*asan*/ as a function of position and prominence (focus). The target words are underlined with the focused items in bold.

(a)	Focused	Q	ipʌne p*opɨn tanʌnɨn, *[*_*IP*_ ***tasan*** *ap*^*h*^*e nohnajo]*? ‘Shall I put this card in front of ***tasan***?’
IP-initial		A	*aniyo*. *ipʌnen*, *[*_*IP*_ ***t***^***h***^***asan*** *ap*^*h*^*e nohajo]*. ‘No, put it in front of ***t***^***h***^***asan*** this time.’
(b)	Unfocused	Q	ipʌne p*opɨn tanʌnɨn, *[*_*IP*_ *t*^*h*^*asan* ***ap***^***h***^***e*** *nohnajo]*? ‘Shall I put this card **in front of** *t*^*h*^*asan*?’
		A	*aniyo*. *ipʌnen*, *[*_*IP*_ *t*^*h*^*asan* ***twie*** *nohajo]*. ‘No, put it **behind** *t*^*h*^*asan* this time.’
(c)	Focused	Q	ipʌne p*opɨn tanʌnɨn, *[*_*IP*_ *nolan* ***tasan*** *ap*^*h*^*e nohnajo]*? ‘Shall I put this card in front of yellow ***tasan***?’
IP-medial		A	*aniyo*. *ipʌnen*, *[*_*IP*_ *nolan* ***t***^***h***^***asan*** *ap*^*h*^*e nohajo]*. ‘No, put it in front of yellow ***t***^***h***^***asan*** this time.’
(d)	Unfocused	Q	ipʌne p*opɨn tanʌnɨn, *[*_*IP*_ *nolan t*^*h*^*asan* ***ap***^***h***^***e*** *nohnajo]*? ‘Shall I put this card **in front of** yellow *t*^*h*^*asan*?’
		A	*aniyo*. *ipʌnen*, *[*_*IP*_ *nolan* *t*^*h*^*asan* ***twie*** *nohajo]*. ‘No, put it **behind** yellow *t*^*h*^*asan* this time.’

With respect to boundary manipulation, an IP boundary was induced by adding an adverb ‘ipʌnen’(‘this time’) just before the target word (as written in Table 2A and 2B). The speakers indeed placed an IP boundary after the adverb most of the time. To induce the IP-medial condition, the additional adjective ‘nolan’ (‘yellow’) was inserted just before the target word (as written in Table 2C and 2D). As shown in Fig 1, the adjective ‘nolan’ (‘yellow’) was indicated by a yellow background on the board. Given that speakers usually put an IP boundary after the adverb (‘this time’), they grouped the adjective ‘nolan’ (‘yellow’) with the following target word, so that the position of the target stops became IP-medial (the IP-medial condition). This IP-medial condition also caused speakers to produce the target stops in an AP-medial position since they did not put an AP boundary between the adjective (‘yellow’) and the target word. (See Section 2.4 for further discussion on defining IP-medial contexts.)

The prompt questions were pre-recorded by four speakers of Seoul Korean, consisting of two young (male, age 23; female, age 24) and two older speakers (male, age 56; female, age 55). The participants then heard all four versions of each question with an equal number of each version, so that their production would not be biased by or accommodated to a particular speaker’s voice.

Prior to actual recording, the participants had a practice session of about 15 minutes. They went over the list of all the target words along with an example sentence for each word to familiarize themselves with the words. They then practiced producing the target words in the actual carrier sentences. During the practice session, they saw the full text for the mini dialogue along with the information provided on the board. Once they went through the entire list across different conditions, they reported that it would be easy to produce the target sentence without the written text.

The entire test set was repeated twice in a randomized order. When participants produced the answer sentence with obvious mispronunciation or deviant rendition, they were asked to repeat the sentence again. A total of 4096 tokens (32 speakers × 16 target words × 2 boundary positions × 2 focus types × 2 repetitions) were obtained. The prosodic renditions of the obtained utterances were further checked by all three authors, who were trained Korean ToBI transcribers, especially with respect to the boundary type and the focus realization, following the conventions discussed by [23]. We excluded 409 tokens (10% of data) that were realized with inadequate prosodic patterns or that contained heavy glottalization or devoicing on the following vowel, which would make acoustic measurement difficult. The inadequate prosodic patterns were identified as such when speakers mistakenly emphasized a target word which was meant to be unfocused, and when speakers inserted a pause or lengthened the preceding word, creating a percept of an IP juncture before a target word which was intended to be in an IP-medial position.

### 2.3. Measurements

F0 was measured at the time points of 25% and 50% (V-mid point) relative to the entire vowel following the stop. F0 values were first extracted using a Praat script (65–500 Hz; 10 ms time step) and then manually checked one by one based on visual inspection of the pitch contour for each token to avoid F0 measurement errors that might arise with pitch halving/doubling or local pitch perturbation that deviates from the general F0 contour. F0 values in Hz were converted into semitones (St) using the formula 12[log_2_(Hz/100)] with a reference F0 of 100 Hz. We initially considered using measurements based upon the tonal peaks for possible low and high tonal targets, but decided to use two relative time points, namely the 25% and V-mid points for the following reasons. First, a preliminary examination of the data indicated that speakers would generally differ in the exact use of F0 in terms of its segmental anchoring timings for each assumed tonal target (the F0 peak for the H tone and the F0 valley for the L tone). Second, it was often difficult to pinpoint the exact F0 peak and valley during the following syllable, even in the IP-initial condition in which AP-initial tonal targets (H vs. L) are supposed to be clearly defined during the first syllable. Third, identifying the tonal targets in the phrase-medial position was even more difficult because the use of F0 for segmental distinction can be overridden by the AP-internal intonational structure.

VOTs of the target lenis /t/ and aspirated stops /t^h^/ were measured from the point of stop release to the voice onset of the following vowel, defined as the onset of the first formant (F1) seen in spectrograms. For the IP-medial target stops, to take into account voicing during stop closure, we measured both positive VOTs and %-voicing-in-closure (which was defined as the percentage of the voiced interval during the closure relative to the entire closure duration).

### 2.4. Defining the IP-medial position in the focused condition

It is worth noting here that [25, 34] suggests that the prominence in Seoul Korean may be inseparable from the structural effect, so that the presence of focus may induce a kind of phrase-level prosodic boundary [22, 35]. specifically suggested that focus initiates an Accentual Phrase (AP). This claim was made based on the phonetic evidence of an intonational pattern of LH on a focused word. The initial LH pattern has been assumed to be important phonological evidence for an AP in the theory of Korean intonational phonology that has been developed by Jun ([22, 23, 25]). If this were the case, the focus-related effect could be interpreted simply as the boundary-related effect. Thus, a word in the focused condition would unequivocally be assumed to form an AP, so that a focused word cannot be positioned AP-medially.

**Fig 2:**
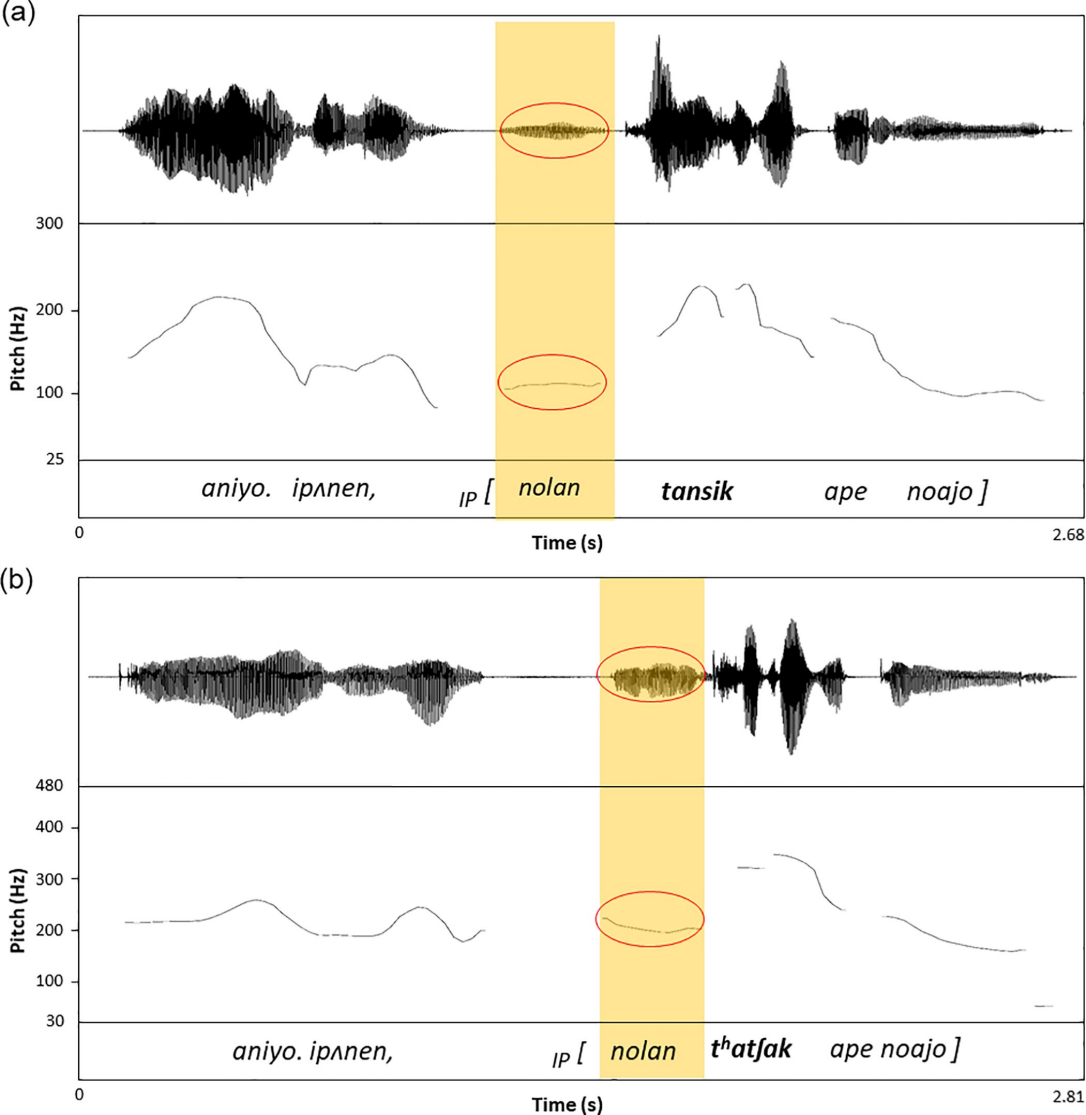
Example waveforms of sentence tokens along with F0 realization in the IP-medial condition. The target words are in bold under focus (a) with a lenis stop in *tansik* (‘fast’) and (b) with an aspirated stop in *t*^*h*^*atʃak* (‘threshing’). In both (a) and (b), the highlighted portions of the pre-focal word *nolan* (‘yellow’) indicate that they are phonetically reduced along with tonal evidence of its dephrasing, so that it is parsed into an Accentual Phrase (embedded in an IP) together with the following focused words.

Our production data, however, indicate that the presence of focus does not automatically form an AP for the following reasons. First, we found no boundary-related perceptual cues that supported the focus-induced boundary hypothesis. In particular, the focused condition in the IP-medial position was not accompanied by a qualitative percept of an AP juncture between the prefocal word and the focused target word in the temporal dimension. All three authors, who were trained Korean prosodic transcribers, concurred that the pre-focal word was extremely reduced in both the temporal and tonal dimensions, so that there was no percept of an AP juncture at all between the pre-focal word and the focused word. As can be inferred from examples in Fig 2, there was no sign of preboundary lengthening at all but rather the pre-focal word perceived to be extremely reduced showing a pre-accentual shortening. Second, the tonal pattern of the pre-focal word was flattened, indicating that it was ‘dephrased.’ The phonetic reduction and dephrasing of a pre-focal word is exemplified in Fig 2. It is also important to note that the focused word *tansik* in Fig 2A does not start with a tone lower than the preceding F0 of the pre-focal word, which is an additional piece of evidence that the focused word does not initiates an AP. As can be seen from the highlighted portions in the waveform in the figure, the pre-focal word *nolan* (‘yellow’) in both the lenis and the aspirated context was substantially reduced in both duration and amplitude along with a flattening of tone. Such multidimensional phonetic reduction indicates clearly that the pre-focal word is dephrased, and must be parsed into a phrase together with the following focused word, as stipulated by the Strict Layer Hypothesis ([36]; also [14] for related discussion). In fact [35], also noted that the pre-focal sequence tends to be shorter than that in a neural sentence, but no theoretical development has been made with respect to how a phonetically reduced (and possibly dephrased) pre-focal word must be parsed in the hierarchy of prosodic structure. In other words, if the focused word initiates an AP, its left edge must be aligned with the left edge of an AP, and the right edge of the preceding word be aligned with the right edge of an AP. But when the reduced pre-focal word is not aligned with the right edge of an AP, it can be interpreted as part of a phrase together with the focused word. One might argue that the reduced element may in theory be treated as being extra-metrical, violating the assumptions of the Strict Layer Hypothesis. We leave this issue open for further research, but for the purpose of the present study, we assume that the reduced pre-focal word must be parsed into an AP along with the focused word, so that the focused word alone does not form an AP. Thus, the IP-medial condition in the present study is the same as the AP-medial condition.

### 2.5. Statistical analyses

Statistical analyses were carried out with R 3.5.1 [37] using linear mixed-effects (lme) models for each acoustic measure (i.e., VOT, %-voicing-in-closure, F0 at the 25% and V-mid points). The *lmer* function from the *lmerTest* package was used to calculate the models [38]. The analyses were carried out separately for each prosodic position (i.e., IP-initial & IP-medial) because, as discussed in the introduction, interactions between phonetics and prosody would differ in IP-initial and IP-medial positions (e.g., the position-dependent lenis stop voicing rule and the segment–tone interaction), which would make it difficult to compare the results directly between the IP-initial and IP-medial conditions. The independent variables were coded using a sum contrast coding: C-type (0.5: aspirated, −0.5: lenis), Focus (0.5: focused, −0.5: unfocused), Gender (0.5: female, −0.5: male), and Age (0.5: younger, −0.5: older). (Note that the weights were adjusted to take into account different numbers of observations between the levels in each factor, so that the actual values were not precisely 0.5 and -0.5. The actual values used for each model are provided in Table 4 in the S1 Appendix.) These factors were included as fixed effects along with all interactions on each of the three dependent variables (VOT, %-voicing-in-closure, F0). In addition, F0 was taken at two points in the vowel which were likely to be autoregressive (as pointed out by a reviewer), but it is possible that the tonal pattern changes substantially from the 25% timepoint to the V-mid point as a function of whether the initial tone is L or H. Thus, given that the effect of Timepoint was not of theoretical interest for the purpose of the present study, Timepoint was just added as a control factor (with a sum contrast coding; 0.5: 25%, -0.5: midpoint) to control for variance that may be introduced by Timepoint (e.g., [39]).

Whenever there was a significant interaction between C-Type and any other fixed-effect factor, we fitted a subset of the data into separate lme models to further examine the C-type effect in each condition. For example, when there was an interaction between C-type and Age on VOT, we ran lme models with C-Type as a fixed effect separately in the young and the old speaker groups, so that we could test whether the C-type effect on VOT would still be significant in each speaker group separately. Finally, we tested the effect of C-type on each dependent measure in each condition separately by Age, Gender and Focus, which illustrated a possible four-way interaction pattern. The results were reported for the sake of completeness regardless of whether the four-way interaction was significant or not.

All models included possibly maximal random effects structures (i.e., random intercepts and slopes for speakers and items) to the extent that they were supported by the data. In cases of non-convergence, the random effects structure was simplified by eliminating the by-item random slope first. This decision was made on the basis of the magnitude of variance, which was smaller for the by-item random effects than for the by-speaker random effects. (See Table 5 in S1 Appendix for the exact R formula used for linear mixed effects models.)

## 3. Results

In this section, we will report results focusing on the effect of C-type (lenis vs. aspirated) and its interaction with the other factors of Age, Gender, and Focus. The relevant results will be summarized in figures. Results of all linear mixed-effects models for all effects and interactions are provided in Tables 6, 7 in the S1 Appendix.

### 3.1. Variation of VOT and F0 in IP-initial position

#### 3.1.1. Variation of VOT in IP-initial position

**Fig 3:**
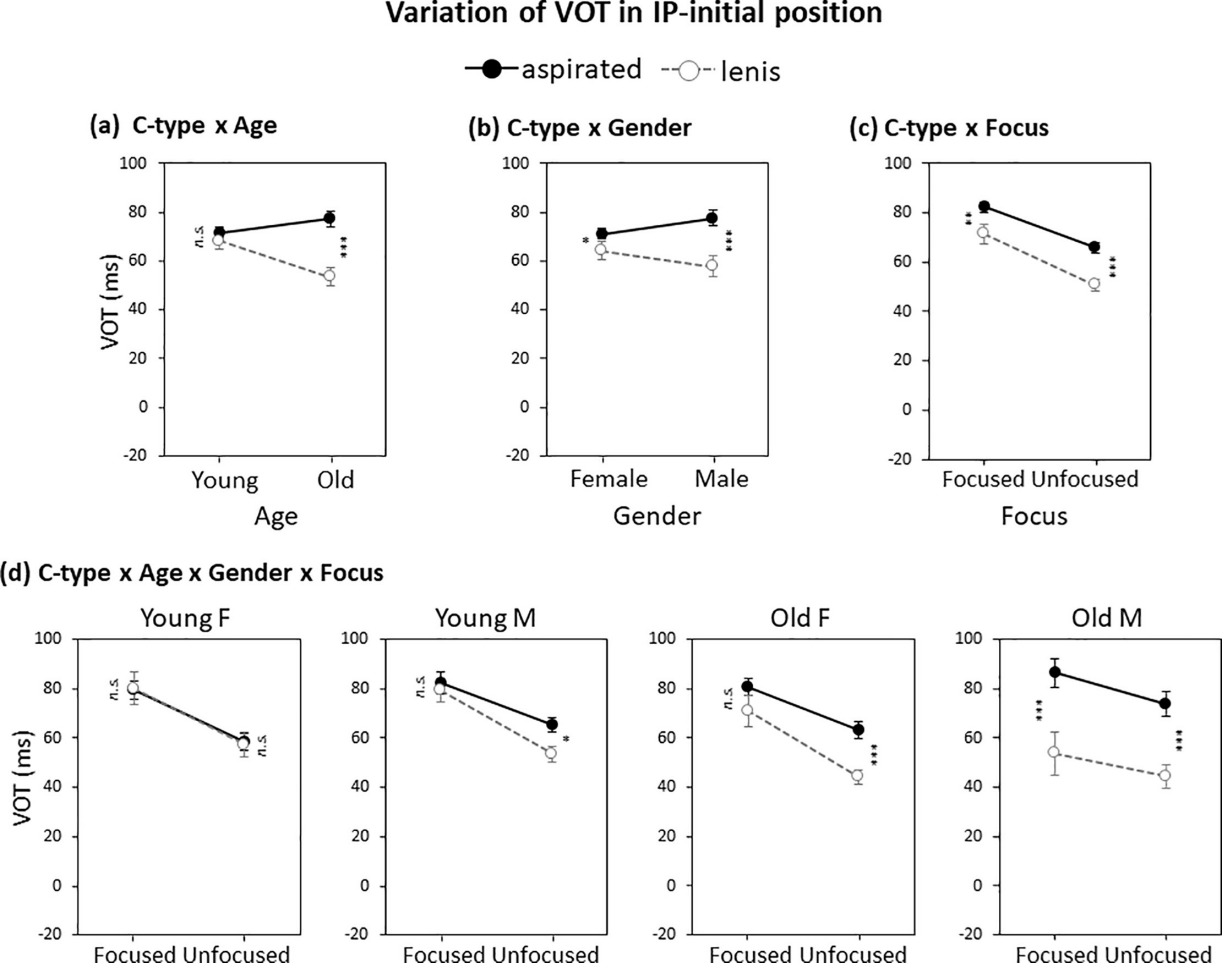
Variation of IP-initial VOT by (a) C-type × Age, (b) C-type × Gender, (c) C-type × Focus and (d) C-type × Age × Gender × Focus. Error bars show standard errors. *p < 0.05; **p < 0.01; ***p < 0.001; *n*.*s*., not significant.

There was a significant main effect of the C-type factor (*β* = 12.7, *p*<0.001). Crucially, however, the C-type effect on VOT interacted with Age and Gender, respectively (C-type × Age, *β* = −18.7, *p*<0.001; C-type × Gender, *β* = −13.0, *p*<0.001). As can be seen in Fig 3A and 3B, the age-related interaction stemmed from the fact that the lenis–aspirated distinction was significant only for the old group (young, *β* = 3.4, *p*>0.1; old, *β* = 22.9, *p*<0.001), and the gender-related interaction from the fact that the use of VOT was more robust in the male group than in the female group (male, *β* = 19.9, *p*<0.001; female, *β* = 5.7, *p*<0.05). It was also noticeable that the reduction of VOT use for the lenis–aspirated distinction by the young group (vs. the old group) and by the female group (vs. the male group) came from both sides, in that the VOT was shortened for the aspirated stop but lengthened for the lenis stop. But there was no three-way interaction of C-type × Age × Gender (*β* = 11.1, p>0.1), indicating that the age- and gender-related interaction effects were independent of each other.

As for the effect of Focus (prominence), there was also a significant main effect of the Focus factor (*β* = 18.8, *p*<0.001), with VOT being longer in the focused than in the unfocused condition. Crucially, as can be inferred from Fig 3C, there was no C-type × Focus interaction (*β* = −4.2, *p*>0.1), indicating that the overall use of VOT for the lenis–aspirated distinction did not differ as a function of focus and hence there was no general enhancement of the lenis–aspirated distinction under focus. In other words, the laryngeal distinction as reflected in VOT was not generally enhanced under focus. Furthermore, the focus factor was not involved in any other three-way interactions with C-type, age, or gender.

There was, however, a marginal four-way interaction that involved all four factors (*β* = 19.2, *p* = 0.08). Fig 3D illustrates the results of separate lme models with C-type effects for each focus condition, indicating how the lenis–aspirated distinction (C-type effect) may interact with Focus across four different groups separated by age and gender. The young female speakers (Young F) showed no use of VOT at all for the lenis–aspirated distinction, with the general focus effect of increasing VOT for both consonant types. The young male speakers (Young M) showed the use of VOT only in the unfocused condition. The old female speakers (Old F) showed a similar pattern as the young male speakers (Young M), showing the use of VOT only in the non-prominent (unfocused) condition, although the old female speakers (Old F) tended to use VOT more than the young male speakers (Young M). Finally, the old male speakers (Old M) showed the most remarkable use of VOT across the board, with a clear-cut separation of the lenis and the aspirated stops by means of VOT in both the focused and the unfocused conditions. Interestingly, unlike the old female speakers (Old F) who showed no consonant effect in the prominent (focused) condition, the old male speakers (Old M) increased the VOT difference in the focused condition in a direction towards an enhancement of the laryngeal contrast under focus. Cross-sectional patterns across different age and gender groups (as shown in the four panels of Fig 3D) indicated the following trend in the possible ongoing sound change in the use of VOT: Young F (no use) < Young M < Old F < Old M (full use).

#### 3.1.2. Variation of F0 in IP-initial position

**Fig 4:**
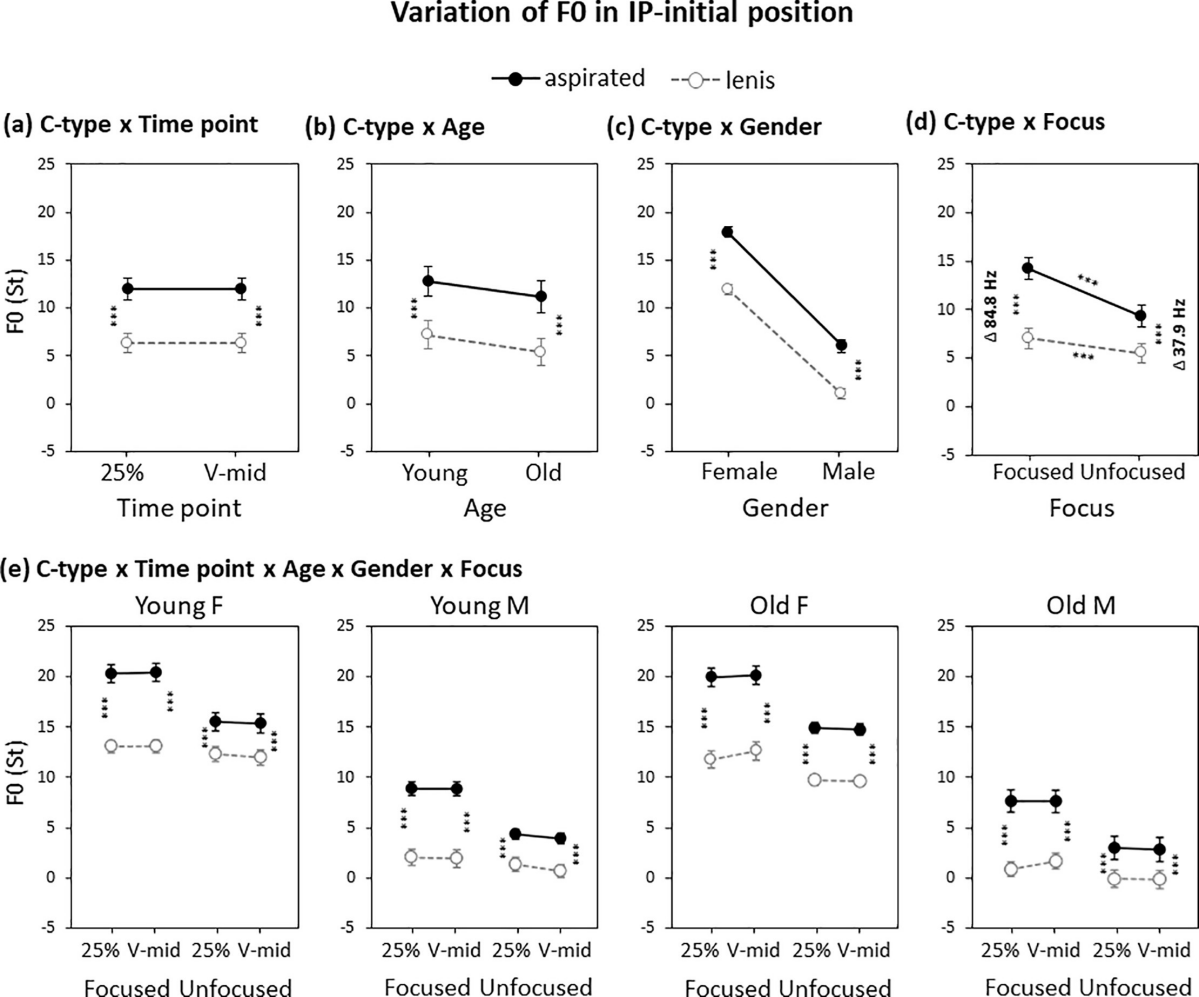
Variation of IP-initial F0 by (a) C-type × Timepoint, (b) C-type × Age, (c) C-type × Gender, (d) C-type × Focus and (e) C-type × Age × Gender × Focus × Timepoint. Note that in b–d, the F0 (St) values over the two timepoints are averaged. Error bars show standard errors. ***p < 0.001.

There was a significant main effect of C-type on F0 (*β* = 5.5, *p*<0.001), with F0 being higher for the aspirated than for the lenis stops. The effect of C-type on F0 did not interact with the Timepoint factor during the vowel (25% and V-mid), showing a general consonant effect on F0 spreading into the midpoint of the vowel (Fig 4A). C-type also interacted with Gender (*β* = 1.2, *p*<0.05), with the effect of the C-type factor being greater in the female than in the male speaker group (Fig 4C), but C-type did not show a two-way interaction with Age (Fig 4B), indicating that the use of F0 for the lenis–aspirated distinction was largely comparable across the young and old speaker groups. Interestingly, however, there was a three-way interaction of C-type × Age × Timepoint (*β* = -0.6, *p*<0.001), which was due to the age-dependent difference in whether the F0 difference increases or decreases into the midpoint of the vowel. In the young speaker groups, the difference in F0 for the lenis–aspirated distinction was larger at the V-mid point than at the earlier (25%) timepoint of the vowel, indicating that the use of F0 goes beyond a segmental perturbation effect. In the old speaker groups, on the other hand, the reverse was true, so that the difference in F0 was smaller at the V-mid point than at the 25% timepoint. This indicates that variation of F0 still reflects some degree of the segmental perturbation effect in the production of the old speakers.

Finally, the C-type factor interacted with the Focus factor (*β* = 3.5, *p*<0.001), which was due to the fact that the effect of the C-type factor was larger in the focused than in the unfocused condition, showing a kind of enhancement of the lenis–aspirated distinction under focus. The focus-induced enhancement was attributed to an increase of F0 primarily for the aspirated stop under focus, whereas the increase of F0 for the lenis stop was minimal (Fig 4D). Importantly, note that the F0 for the lenis stop did not *decrease* under focus, indicating that the direction was not an enhancement of the target of the L tone in particular. Analysis of the C-type × Focus interaction for each group separated by age and gender confirmed that the use of F0 for the lenis–aspirated distinction is largely comparable across groups, even though it might be redundant for the old male speaker group who showed a clear-cut VOT use for the lenis–aspirated distinction (Fig 4E).

### 3.2. Variation of voicing and F0 in IP-medial position

#### 3.2.1. Variation of VOT in IP-medial position

**Fig 5:**
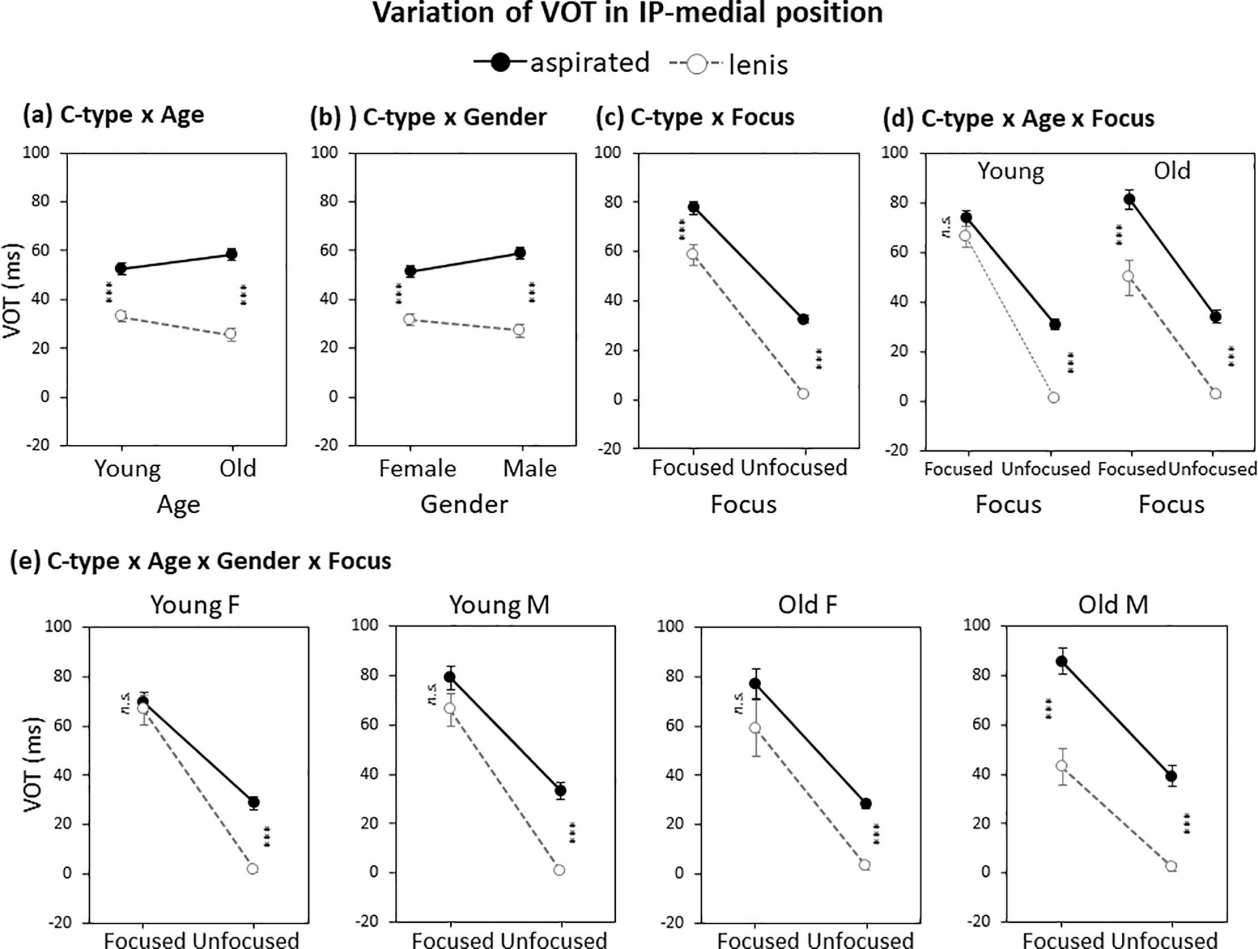
Variation of IP-medial VOT by (a) C-type × Age, (b) C-type × Gender, (c) C-type × Focus, (d) C-type × Age × Focus, and (e) C-type × Age × Gender × Focus. Error bars show standard errors. ***p < 0.001; *n*.*s*., not significant.

There was a significant main effect of C-type (*β* = 24.2, *p*<0.001), showing a substantial use of VOT for the lenis–aspirated distinction in phrase-medial positions (Fig 5). C-type showed a significant two-way interaction with Age and Gender, respectively. As can be seen in Fig 5A and 5B, the difference in VOT used for the lenis–aspirated distinction in the phrase-medial position was generally smaller for the young than for the old speaker group (Fig 5A, C-type x Age, *β* = -10.2, *p*<0.05); and also generally smaller for the female than for the male speaker group (Fig 5B, C-type x Gender, *β* = -14.3, *p*<0.001). As far as the two-way interactions of C-type with Age or Gender were considered (i.e., when the data were pooled across focus conditions), unlike the phrase-initial use of VOT which was not observed in the young speaker group and extremely reduced in the female speaker group, the phrase-medial lenis–aspirated distinction was still maintained in VOT regardless of Age and Gender—i.e., in both the young and the old speaker groups and in both the female and the male speaker groups. (But see below for further interactions with Focus, which indicates that this across-the-board effect is largely limited to the unfocused condition).

As for focus-related prominence effects, there was a two-way interaction of C-type x Focus (*β* = -13.0, *p*<0.01) and as well as a three-way interaction of C-Type x Age x Focus (*β* = -18.2, *p*<0.05). While there was a reduced VOT difference in the focused condition as compared to that in the unfocused condition (Fig 5C, C-type x Focus), the three-way interaction of C-type × Age × Focus, as can be seen in Fig 5D, stemmed from the fact that while both the young and the old speaker groups used VOT for the phrase-medial lenis–aspirated distinction in the non-prominent (unfocused) condition, the young speaker groups no longer used VOT in the prominent (focused) condition. The old speaker group still showed a significant effect of C-type on VOT in this prominent (focused) condition (right panel of Fig 5D; *β* = 28.6, *p*<0.001).

There was no four-way interaction among factors nor was there any other significant interaction. But it is noteworthy that the old male speakers (Old M) showed the exact opposite of what the other groups showed. As can be seen in Fig 5E (the rightmost panel), only the old male speakers (Old M) showed an augmented difference in VOT under focus, showing a tendency towards a focus-induced enhancement of the contrast of the laryngeal feature. Again, the cross-generational variation of VOT across the gender groups in the IP-medial position presents a direction of the possible ongoing sound change in use of VOT comparable to that in the IP-initial position insofar as the prominent (focused) condition is concerned: Young F (no use) < Young M < Old F < Old M (full use). But in the non-prominent (unfocused) condition, speakers of all four groups used VOT, which could be accounted for by the substantially reduced VOT due to lenis stop voicing only in this prosodically weak context.

#### 3.2.2. Variation of %-voicing-in-closure in IP-medial position

To examine the lenis stop voicing effect, we first checked how many tokens of the lenis stops were realized as ‘substantially’ voiced in the intervocalic phrase-medial position in accordance with the lenis stop voicing rule. We counted tokens as ‘substantially’ voiced if there was acoustic evidence for voicing observed in the spectrogram and the waveform in more than 50% of the closure. This 50% criterion of voicing during closure was suggested by [40] (cf. [41]) as a threshold to classify phonetically voiced variants into ‘fully’ voiced and ‘partially’ voiced ones, although ‘fully’ voiced tokens have often been considered in the phonetics literature as containing phonetic voicing for the most part of the closure (e.g., [20, 42]). As given in Table 3, the occurrence of the lenis stop voicing varied depending on the focus-induced prominence factor. In the non-prominent (unfocused) condition, the lenis stops were produced as voiced most of the time across the groups (93% on the average) in accordance with the lenis stop voicing rule. But the opposite was true in the prominent (focused) condition, even in the IP-medial (at the same time AP-medial) position, so that only 7% of the lenis stop tokens were produced as voiced, similar to the occurrence of voiced tokens for the aspirated stops which do not undergo lenis stop voicing. These results indicate that the application of the lenis stop voicing rule is further modulated by the prominence factor in addition to the boundary-related condition (i.e., AP-medial) in such a way that the lenis stop voicing is blocked under focus.

**Table 3:** Numbers (proportions in parentheses) of stops that were realized as ‘substantially’ voiced (with more than 50% of voicing during closure) in IP-medial positions, as a function of consonant type, focus, and speaker group. (Note that the ‘substantially’ voiced tokens may be considered to be ‘fully’ voiced if we apply the threshold of 50% of voicing during closure as suggested by [40]).

		Lenis		Aspirated	
		Voiced	Voiceless	Voiced	Voiceless
Focused	Young F	6 (5%)	119 (95%)	5 (4%)	122 (96%)
	Young M	1 (1%)	115 (99%)	2 (2%)	109 (98%)
	Old F	13 (13%)	85 (87%)	4 (4%)	107 (96%)
	Old M	14 (11%)	110 (89%)	7 (6%)	111 (94%)
	Mean	34 (7%)	**429 (93%)**	18 (4%)	**449 (96%)**
Unfocused	Young F	110 (87%)	16 (13%)	18 (15%)	100 (85%)
	Young M	125 (98%)	3 (2%)	14 (12%)	107 (88%)
	Old F	107 (92%)	9 (8%)	10 (10%)	94 (90%)
	Old M	115 (93%)	9 (7%)	32 (28%)	83 (72%)
	Mean	**457 (93%)**	37 (7%)	74 (16%)	**384 (84%)**
Overall		491 (51%)	466 (49%)	92 (10%)	833 (90%)

**Fig 6:**
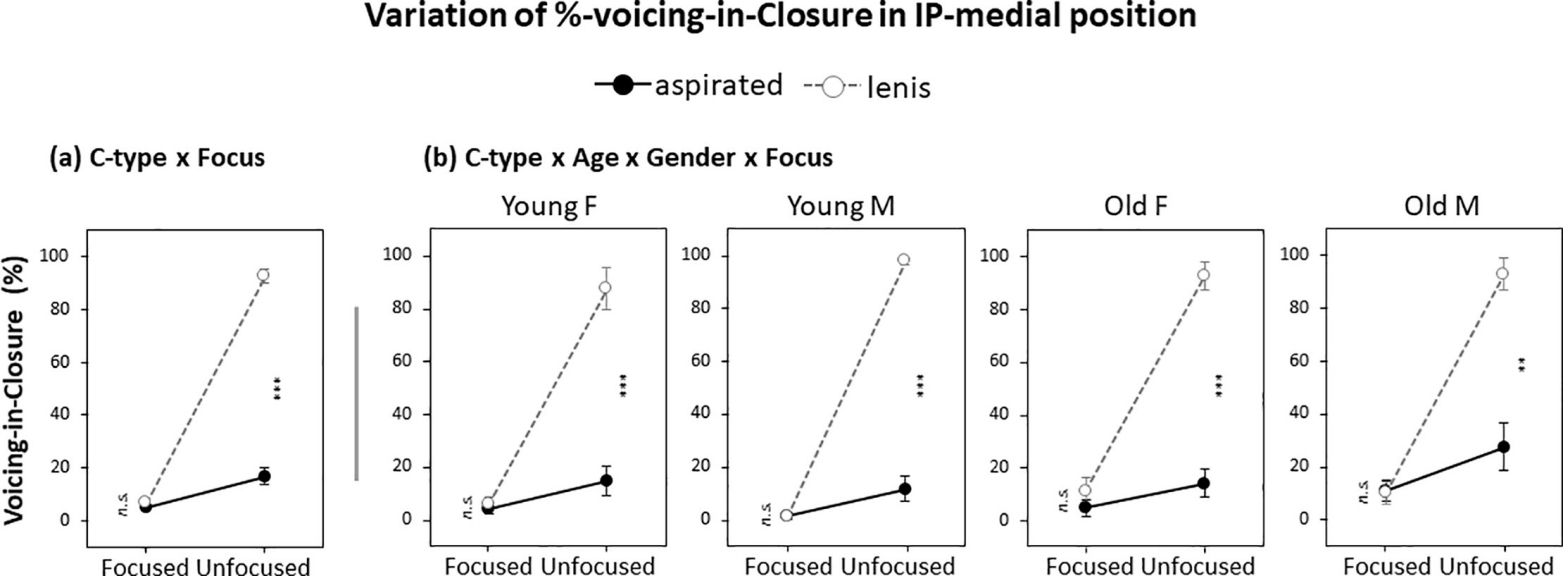
Variation of %-voicing-in-closure in IP-medial position by (a) C-type x Focus and (b) C-type x Age x Gender x Focus. Error bars show standard errors. ** *p* < 0.01; ***p < 0.001; *n*.*s*., not significant.

Results of LME modeling carried out on %-voicing-in-closure were largely consistent with these observations. There was a significant main effect of C-type on %-voicing-in-closure (*β* = -39.5, *p*<0.001), showing more voicing during closure for the lenis than for the aspirated stops in phrase-medial positions (lenis, mean 51%, sd, 49%; aspirated, mean 11%, sd, 28%). But unlike the effect on VOT, there was no effect of Age and Gender, nor was there any interaction of C-type with either Age or Gender (see Table 6 in S1 Appendix for the statistical details). In other words, the difference in %-voicing-in-closure used for the lenis-aspirated distinction was not influenced by Age or Gender in any ways. There was only one significant interaction among factors; that is, C-type and Focus (*β* = 76.4, *p*<0.001) while no other significant interactions among factors were observed. As can be seen in Fig 6A, the C-type x Focus interaction was due to the fact that the C-type effect on %-voicing-in-closure was significant only in the non-prominent (unfocused) condition, regardless of Age and Gender, and the effect disappeared in the prominent (focused) condition even in the phrase-medial position. This prominence-dependent C-type effect on %-voicing-in-closure can be seen in Fig 6B across the four different speaker groups (by Age and Gender).

#### 3.2.3. Variation of F0 in IP-medial position

**Fig 7:**
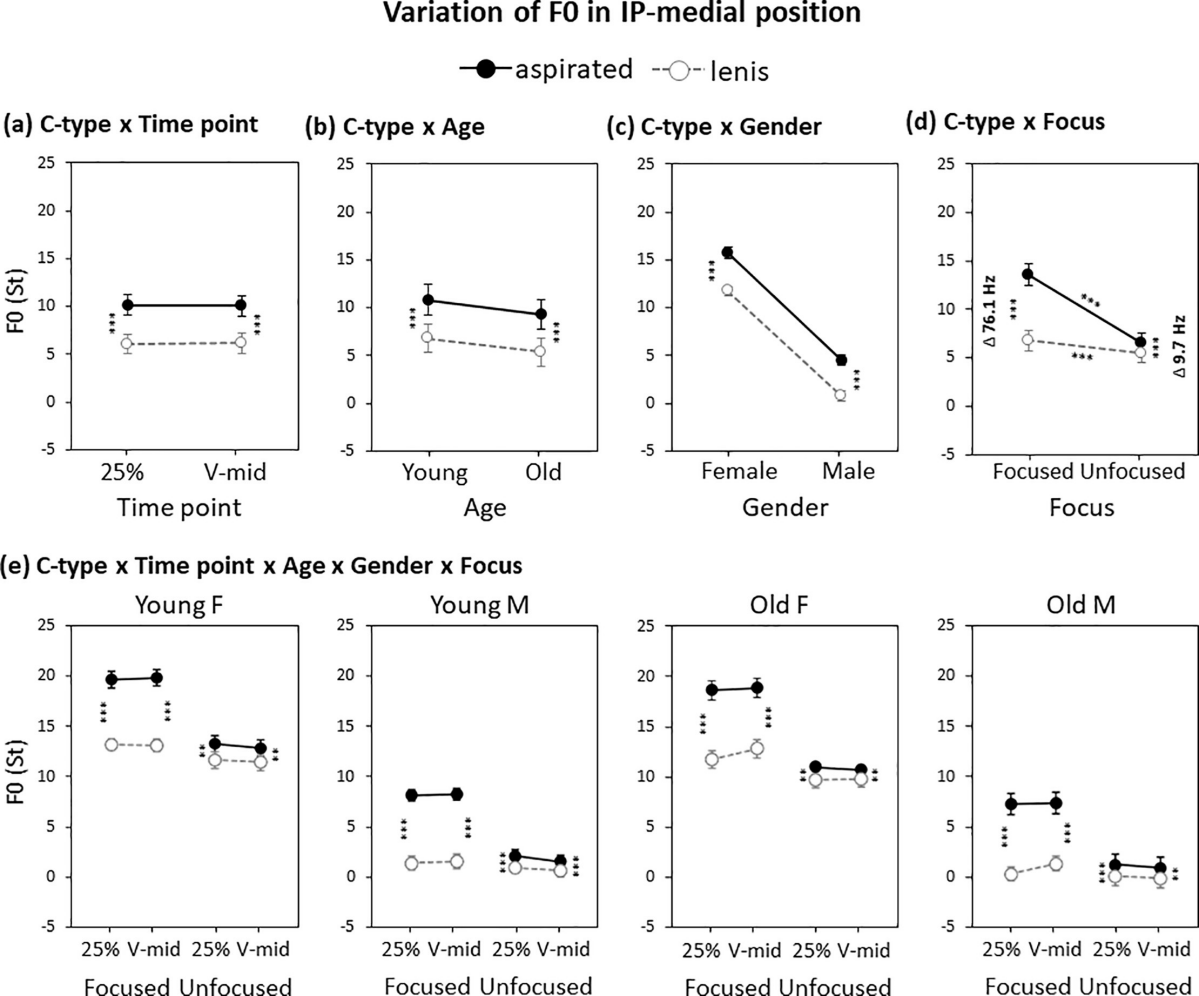
Variation of IP-medial F0 by (a) C-type × Timepoint, (b) C-type × Age, (c) C-type × Gender, (d) C-type × Focus and (e) C-type × Age × Gender × Focus × Timepoint. Note that in b–d, the F0 (St) values over the two timepoints are averaged. Error bars show standard errors. **p < 0.01; ***p < 0.001.

There was a significant main effect of C-type upon F0 in the IP-medial position (*β* = 3.8, *p*<0.001), with F0 being higher for the aspirated than for the lenis stop, showing a general phrase-medial use of F0 for the lenis–aspirated distinction. C-type also interacted with timepoint (*β* = 0.2, *p*<0.05), but results of separate lme models built at each time point revealed that the use of F0 for the lenis–aspirated distinction did not differ much between the 25% and V-mid points (mean diff. 4.1 St at 25%; mean diff. 3.9 St at V-mid; see also Fig 7A). C-type did not interact with either Age (*β* = 0.2, *p*>0.1) or Gender (*β* = 0.2, *p*>0.1), indicating that the use of F0 for the lenis–aspirated distinction was largely comparable across the Age and Gender factors (Fig 7B and 7C). C-type, however, interacted with Focus (*β* = 5.3, *p*<0.001). The C-type × Focus interaction was attributable to the F0 difference being much larger in the focused than in the unfocused condition (Fig 7D). As was the case for the IP-initial position, the focus-induced enhancement of the F0 difference stemmed primarily from the larger magnitude of the F0 increase for the aspirated stop, whereas the F0 increase for the lenis stop was minimal. Again as in the phrase-initial case, F0 for the phrase-medial lenis stop did not decrease under focus, showing no direct enhancement of the L tone. Fig 7E shows the C-type × Focus interaction for each group separated by age and gender; as in the IP-initial position, the use of F0 for the IP-medial lenis–aspirated distinction was largely comparable across the board with an augmented F0 difference under focus. This focus-induced enhancement of F0 was observed even for the old male speaker group (Fig 7E, rightmost panel) who also showed a clear-cut use of the segmental voicing feature of VOT for the lenis–aspirated distinction.

Finally, it is important to note that the phrase-medial reduction of the F0 difference in the non-prominent (unfocused) condition was substantially different from the phrase-initial reduction pattern in the same non-prominent (unfocused) condition. While the mean F0 difference for the phrase-initial lenis-aspirated distinction was reduced from 84.8 Hz to 37.9 Hz in the focused vs. the unfocused condition (Fig 4D), the difference in the phrase-medial position was reduced to a greater extent from 76.1 Hz to 9.7 Hz (Fig 7D).

## 4. General discussion

In the present study, we analyzed acoustic data on apparent-time speech in present-day Seoul Korean produced by speakers of different age (young/old) and gender (female/male) groups. This study was aimed at examining how different groups of speakers employ the segmental voicing feature (VOT) and the tonal feature (F0) to maintain the lenis–aspirated distinction in different prosodic structural contexts, so that we could understand the nature of the ongoing sound change from a perspective that includes consideration of the phonetics–prosody interface.

### 4.1. Prosodically-conditioned use of the segmental voicing feature (VOT) versus the tonal feature

One of the basic findings of the present study is that the use of the segmental voicing (VOT) feature versus tonal (F0) features for the phonological contrast of the lenis and the aspirated stops varies as a function of age (22 years old vs. 59 years old at the time of recording in 2017) and gender. The present results clearly show that young speakers use VOT less and F0 more than old speakers do (an age effect), and female speakers use VOT less and F0 more than male speakers do (a gender effect), confirming that the ongoing sound change has been led by the young female speakers in the speech community (e.g., [5, 6, 43], cf. [44]). Furthermore, the results newly demonstrate that the use of VOT and F0 is conditioned by prosodic structural factors such as prominence (focused/unfocused) and prosodic position (phrase-initial vs. phrase-medial). As far as the phrase-initial (IP-initial) production data is concerned, the young female speakers show a complete phrase-initial VOT merger; that is, they do not use VOT at all for the lenis–aspirated distinction, regardless of prominence (focused or unfocused). Rather, the young female speakers (Young F) use F0 exclusively for the phrase-initial lenis–aspirated distinction. They also enhance the F0 difference under focus, suggesting that the phonetic content of F0 informs the phonological contrast to some extent (e.g., [11, 27, 30]). This pattern is followed, though to a lesser extent, by the young male speakers (Young M) and the old female speakers (Old F). Both groups (Young M and Old F) do not use VOT phrase-initially, showing a possible VOT merger, but this is true only in the prominent (focused) condition. In the non-prominent (unfocused) condition, the two groups still employ VOT for the phrase-initial lenis–aspirated distinction, indicating that they are at an interim stage in the path of the ongoing sound change. The results further show a difference between these two groups: the difference in VOT is smaller for the young male speakers (Young M) than for the old female speakers (Old F), suggesting that the sound change towards the VOT merger is more advanced in the former than in the latter group. It should be noted that [45, 46] have shown that the VOT merger was attributable more to a decrease in VOT of the aspirated stop rather than an increase in VOT of the lenis stop. Our IP-initial data, however, indicated an opposite trend that the VOT merger by the young group (vs. the old speaker group) (see Fig 3A) and by the female group (vs. the male group) (see Fig 3B) was driven primarily by an increase in VOT of the lenis stop, although VOT of the aspirated stops tended to decrease to a smaller degree. We do not have an explanation to offer, except for noting that there is an 8–10 year gap between [45, 46] and the present study, and the data have been obtained in different prosodic contexts. These two groups, on the other hand, employ F0 fully regardless of prominence (focused or unfocused). They also enhance the F0 difference under focus, indicating that the F0 feature is used substantially for maximizing the phonological contrast under focus.

The old male speakers (Old M), who are generally assumed to be most conservative in sound change (cf. [44]), indeed show preservation of VOT. They use the VOT for the lenis–aspirated distinction in both the focused and the unfocused conditions, and also show a tendency towards an enhancement of the VOT difference under focus. This pattern is an exact opposite of what the other three speaker groups show (no use of VOT under focus). And the tendency towards an increased VOT difference under focus implies that at least in the old male speakers (Old M) the VOT has not lost its feature primacy as a phonologically relevant phonetic feature. Interestingly, however, they also employ F0 in full just like the young female speakers, suggesting that the old male speakers (Old M) employ both of the segmental voicing feature (VOT) and the tonal (F0) feature for maintaining the phonological contrast of the lenis and the aspirated stops.

It is also worth noting here to what extent the focus-induced enhancement effects on F0 vs. VOT observed in the present study may compare to those observed in clear speech [3], especially given that both contexts are likely to induce ‘hyperarticulation’ (localized hyerarticualtion vs. (global) hyperarticulation, respectively) ([3, 26, 47]. Showed that only younger speakers (from 20 to 29 years old, all born before 1977) employed F0 to enhance the lenis-aspirated distinction in clear speech (compared to conversational speech and citation forms), primarily by increasing F0 for the aspirated stop. The younger speakers in [3] also used VOT for the lenis-aspirated distinction only in clear speech. On the other hand, the older speakers in [3] (from 40 to 60 years old, all born before 1966) did not use F0 at all for the lenis-aspirated distinction across different speech styles nor did they show an F0 enhancement for the lenis-aspirated distinction in clear speech. The findings of the present study obtained with the old generation (born between 1954 and 1961) therefore suggests a possible pathway of the sound change indicating that the use of F0 for the lenis-aspirated distinction has newly spread to the old generation over the past 15 years so. Another interesting finding of [3] is that the F0 difference that arises with the segmental distinction of /h/ (higher F0) vs. /n/ (lower F0) did not show an enhancement effect on F0. This suggests that the post-lexically available tones are used for phonological contrasts of segments which are minimally different along the same phonetic dimension (e.g., VOT), but not for segments whose phonological specifications are realized along the entirely different phonetic dimensions (a glottal fricative vs. a nasal stop).

Regarding the phrase-medial position, however, the picture of the differential use of VOT and F0 is skewed further as a function of prominence. The basic use of VOT in the phrase-medial (IP-medial) position, as the results show, is comparable to that observed with VOT in the phrase-initial (IP-initial) position only in the focused condition—i.e., all but the old male speaker group (Old M) do not use VOT for the lenis–aspirated distinction whereas the old male speakers (Old M) tend to enhance the difference in VOT under focus even in the phrase-medial position. Again, in this prominent (focused) condition, all four speaker groups augment the use of F0, showing an F0-induced enhancement of phonological contrast. But the point of divergence from the patterns observed in the IP-initial position is the across-the-board use of VOT in the non-prominent (unfocused) condition; that is, all four speaker groups employ the segmental voicing feature of VOT for the lenis–aspirated distinction. In addition to the use of VOT, all four speaker groups also use F0 for the lenis–aspirated distinction in that position, suggesting that in the non-prominent (unfocused) phrase-medial position, both the segmental and the suprasegmental (F0) features are used for the lenis–aspirated distinction across the board.

Of course, the use of the segmental voicing feature (VOT) in the phrase-medial position is attributable in part to the lenis stop voicing rule, which is modulated by the prosodic structure in which the stop occurs. (Recall that the lenis stop voicing rule in Seoul Korean stipulates that the lenis stop becomes voiced intervocalically when it occurs in a phrase-medial position, specifically in a medial position of the Accentual Phrase). Our phonetic transcription data indicate that the lenis stop voicing rule may be blocked not only by a prosodic boundary (e.g., an IP/AP boundary) as has been assumed in the literature [12], but also by the prominence factor. The voiced tokens occur mostly in the non-prominent *unfocused* condition, indicating the lenis stop voicing rule is blocked by focus, regardless of whether the stops occur in the phrase-initial or phrase-medial (AP-medial) position. This is further confirmed by the significant difference in %-voicing-in-closure for the lenis-aspirated distinction, indicating the phrase-medial lenis stop voicing only in the *unfocused* condition across all the speaker groups. Thus, the cross-generational use of VOT in the *unfocused* phrase-medial position can be taken to have come about as a consequence of application of the lenis stop voicing rule in that context, which results in much lower values of VOT and greater %-voicing-in-closure for the lenis than for the aspirated stops. Interestingly, this particular prosodic context (i.e., the non-prominent (unfocused) phrase-medial position) is precisely where the F0 difference becomes substantially attenuated. The extreme reduction in F0 difference (Δ = about 9.7 Hz) is in fact comparable to a mere low-level phonetic effect rather than a categorical tonal effect (see below for related discussion on the reduced F0 difference in this context). It appears that the attenuated role of the F0 feature in marking the lenis–aspirated distinction in this prosodically weak context is complemented by an increased difference in the segmental voicing feature as reflected in VOT and %-voicing-in-closure, eventually leading to the maintenance of a sufficient phonological contrast between the two stops.

Such positional variation in the use of the segmental voicing features (as reflected in VOT and %-voicing-in-closure) vs. F0 is largely comparable to what has recently been reported with regard to how the two-way stop voicing contrast may be phonetically implemented in Tokyo Japanese [48]. [48] showed that the use of F0 was limited to the stop voicing contrast in a phrase-initial (“post-pausal”) position in which the role of VOT is attenuated, whereas F0 was no longer used in a phrase-medial (especially in a word-medial) position in which phonetic voicing served a sufficient phonetic feature for the stop voicing contrast [48]. Further noted, however, that Tokyo Japanese would not soon follow a similar pathway of sound change that Seoul Korean is currently undergoing [48]. Explained that this would be possibly because the phonological system with a two-way voicing contrast (voiced vs. voiceless) in Tokyo Japanese would leave more room for its phonetic distinction along the VOT dimension as compared to a three-way stop contrast system in Seoul Korean, but also possibly because the pitch-accent system would constrain F0 perturbations. It remains to be seen, as [48] noted, how Tokyo Japanese may change in the use of F0 vs. the phonetic voicing feature in relation to the constraints of the phonological system.

Before moving onto the next discussion point, it is worth considering an alternative account with respect to the prosodic context which may trigger the lenis stop voicing rule in Korean. Korean has often been characterized as a so-called ‘edge-prominence’ language in which the prominence system is inseparable from the prosodic phrasing [25, 34]. Crucially, focus is assumed to induce an AP [22, 35] with the focused word being AP-initial. Under this account, the domain of the lenis stop voicing may still be within an AP, and the lack of the voicing effect under focus can still be interpreted as showing that the lenis stop voicing rule is blocked by an AP boundary which is phonologically inserted by focus. But as we discussed in the methods section, our data indicated that the pre-focal word was extremely reduced in both the temporal and tonal dimensions, indicating that the pre-focal word by itself cannot form an AP and it must be parsed into an AP along with the focused word that follows. Thus, insofar as our data are concerned, the pre-focal word and the focused target word appear to form an AP together, so that the focused target word becomes AP-medial (and at the same time IP-medial). But we do not rule out a possibility that the intonational structure of Seoul Korean may be modified by the prominence factors which may be necessarily accompanied by a prosodic juncture. It remains to be seen how this possibility may be substantiated by more systematic studies on the relationship between focus and prosodic phrasing.

### 4.2. The nature of the ongoing sound change: A prosodic account

As has been discussed thus far, what the present apparent-time study presents unequivocally is that the sort of sound change in progress in Seoul Korean can be better understood in reference to prosodic structure. With the premise that prosody (prosodic structure) is an integral part of speech production and perception (e.g., [13, 14, 49]), the results of the present study provide some new insights into how the ongoing sound change may have been initiated and advanced in relation to the prosodic structural conditioning of phonetic and phonological processes. In what follows, we discuss the probable paths through which the F0 difference may have come into use, in place of VOT, to phonologically contrast the two stops in relation to the phonetics–prosody interface, and propose a prosodic account of the ongoing sound change counter to the tonogenetic account.

#### 4.2.1. Phonologization of the segmental F0 perturbation from microprosody to macroprosody

As discussed in the introduction, the F0 difference in the lenis–aspirated distinction has its origin from the low-level F0 perturbation due to differential laryngeal tension associated with the lenis versus the aspirated stops, resulting in a higher F0 for the aspirated than for the lenis stop (e.g., [1, 7, 50–53]). Seoul Korean presents an interesting case in which the low-level segmental perturbation of F0 at the level of microprosody is phonologized at the level of macroprosody, integrating into the intonational phonology of Seoul Korean (cf. [24]). As a result, an H tone is assigned post-lexically to the AP-initial syllable with the aspirated (and the fortis) consonant, and an L tone with all other consonants including the lenis stop [24, 25] (cf. [51]). This is a case of ‘tonogenesis’ in the sense that the low-level F0 perturbation has developed into tones in the grammar of intonational phonology of Seoul Korean, showing a shift from microprosody to macroprosody. This type of tonogenesis, however, differs from general patterns found in other languages in which the exaggerated F0 difference may be transferred directly into phonological contrasts in the segmental phonology of a given language (e.g., [7, 8, 53]).

#### 4.2.2. Emergence of the use of post-lexically assigned tones for the segmental contrast

Given the tones that have already been introduced in the grammar of the intonational phonology of the language, the next stage in the path of the sound change may be that the speakers learn to use the post-lexical tones specified in the phrase-initial position to distinguish between the lenis and aspirated stops that occur in that position. Since the lenis and the aspirated stops in the phrase-initial position are always accompanied by categorically different L and H tones, respectively, the tonal differences may have become perceptually salient in association with these stops (a prerequisite for an incipient sound change), while the VOT feature has become redundant in this particular prosodic position. Presumably driven by the principle of effort minimization, the young speakers may then have chosen the low-cost option in speech production by utilizing the tones that are invariantly available in the phrase-initial position, while minimizing the use of the redundant segmental feature of VOT, eventually leading to the VOT merger advanced by young female speakers in that prosodic position. The young male speakers (Young M) and the old female speakers (Old F) then follow suit, starting from the prominent (focused) context in which the tonal contrasts are phonetically enhanced and therefore perceptually salient. But in the non-prominent (unfocused) context with no such perceptual saliency of the tonal contrast, the two groups (Young M and Old F) still employ VOT albeit with a gradually diminishing role, showing signs of the VOT merger similar to that found for young female speakers. The VOT merger in this context appears to be more advanced in young male speakers (Young M) than in old female speakers (Old F; recall that the mean VOT difference was smaller for the young male speakers than for the old female speakers). Contrastingly, the most conservative group, old male speakers (Old M), does not seem to have entered the effort minimization process, still preserving both the VOT and tonal features even in the prominent (focused) context where the tonal contrast may be perceptually salient.

#### 4.2.3. Spread of the phrase-initial use of post-lexical tones into other prosodic positions

The question that follows here is then whether the use of phrase-initially specified tones has begun to spread into phrase-medial positions in which there is no segment–tone interaction (i.e., distribution of tones is not determined by the segmental make-ups). A partial support for such a positional carryover in progress comes from the fact that speakers of all four groups show a statistically significant difference in F0 for the lenis–aspirated distinction in the phrase-medial position. But unlike the case for the phrase-initial position, in which the F0 difference is substantially comparable between the focused and the unfocused conditions, the phrase-medial use of F0 is categorically different as a function of prominence (focused/unfocused). In other words, whereas the F0 difference of the lenis–aspirated distinction is similar in its magnitude across positions in the prominent (focused) condition (IP-initial, Δ = 84.8 Hz; IP-medial, Δ = 76.1 Hz), the difference in the non-prominent (unfocused) condition is drastically reduced to 9.7 Hz phrase-medially (IP-initial, Δ = 37.9 Hz; IP-medial, Δ = 9.7 Hz). Note that in the work of [24] the grounds for claiming a phonologization of the microprosodic F0 difference in the intonational phonology of Seoul Korean lay in that the magnitude of the F0 difference (around 50 and 60 Hz) was substantially larger than that of a typical phonetic F0 perturbation effect. The low-level F0 perturbation effects may vary depending on various other factors, but German data reported by [52] indicated an F0 perturbation effect of around 11.6 Hz due to the lax–tense difference; and American English data reported by [54] showed a substantial F0 difference (roughly in the range of 40–80 Hz; see Fig 7 of [54]) due to the stop voicing difference in a relatively prominent (high-pitch) context. Thus, it is reasonable to assume that the small phrase-medial F0 effect (Δ = 9.7 Hz) in the non-prominent (unfocused) context remains at the microprosodic level, driven by the low-level segmental perturbation, rather than by a spread of the phrase-initial ‘phonologized’ tonality effect into the non-prominent phrase-medial position. These observations allude to an interim stage of the prominence-dependent positional carryover of tones whereby the phrase-initial use of post-lexical tones has spread into phrase-medial positions only in the prominent (focused) condition, which may also have modified the intonational structure inside a phrase under focus (see Footnote 1 for related discussion).

Why is then the spread of the use of tones from the initial to the non-initial position constrained by prominence? An answer can be found in connection with the phonetic nature of the speech input that children are exposed to. Infant- or child-directed speech often takes hyperarticulated forms which are produced with prosodic prominence and junctures (e.g., [55–57]). The speech input provided to children is likely to contain stop-initial words produced either in isolation or in phrase-initial position with exaggerated prosody of the L and H tones. The hyperarticulated prosody of child-directed speech may have been an impetus for the extended use of the post-lexical tones as long as the stops occur in the prominent context (i.e., under focus) in which tones are licensed by the intonational phonology, be it phrase-initial or phrase-medial. The extended use of post-lexical tones initiated by young speakers may then have been transmitted to older generations in the speech community. This possibility is indeed in line with what [58] specifically suggest: that prosodic prominence relates directly to the directions and extent of sound change during cross-generational transmission.

#### 4.2.4. The prosodic account over the transphonologization account

The tonogenetic account previously proposed in the literature for the ongoing sound change in Seoul Korean (e.g., [5, 6], cf. [7]) indicates that the ongoing sound change is characterized as transphonologization from VOT to F0 as a primary phonetic feature for a phonological contrast (i.e., the lenis-aspirated distinction). Under this account, one might further assume that the F0 difference due to laryngeal articulations is being ‘transphonologized,’ developing into bifurcated tonal features in the distinctive feature system of the segmental phonology, which goes more or less in parallel with, or perhaps driven by, a diminishing role of the segmental phonetic voicing feature (implemented by VOT). The observations newly reported here and considered in terms of the phonetics–prosody interface lead us to depart from this kind of transphonologization account and to propose instead a prosodic account. The prosodic account is not new in that it is based on phonologization of the segmental F0 perturbation from microprosody to macroprosody as has been proposed in the literature [12, 24]. As we have outlined above, a new tenet of the prosodic account is that the ongoing sound change in present-day Seoul Korean is best characterized as prosodic-structurally conditioned differential use of the segmental voicing feature (i.e., VOT) versus the post-lexically available tones as part of the intonational phonology of the language.

The ongoing sound change in present-day Seoul Korean is understood as a shift from use of the segmental voicing feature to that of the post-lexical tones to the extent that the tones are available in particular prosodic contexts (phrase-initial or focused). The use of post-lexical tones may have spread to the non-initial position provided that the focus-induced prominence attracts the tones, which may re-organize the tonal distribution under focus as part of the sound change. In this account, the ongoing sound change may be tonogenetic in the sense that speakers have begun to utilize the post-lexical *tones* available for the lenis–aspirated distinction in place of VOT, but it is not tonogenetic in the phonological feature system, so that tones are *not* actually being newly transphonologized as distinctive features in the segmental phonology of the language. In other words, if the tones were indeed transphonologized across the board, so that they served as primary cues to segmental contrasts in the way that other phonetic features used for segmental contrasts would be phonetically implemented, we would have found the sufficient tonal contrast regardless of position and prominence. It is, however, worth noting that [8] explored the tonogenetic sound change in Afrikaans only for utterance-initial stops, and noted that it would remain to be determined whether the observed effect would generalize to other contexts [6]. also noted that “[f]or Seoul Korean to develop into a true tonal language, where its lexical items are specified and distinguished by a paradigmatic set of more than one contrastive pitch, the use of contrastive f0 would need to descend from phrase-initial position to lower prosodic levels, for example through the process of ‘domain-narrowing’……Only time will tell whether Seoul Korean will follow this pathway to develop lexical tone” (p.141). These studies appear to imply that in an optimal case a tonogenetic sound change may eventually spread to various other contexts across the board. As reviewers pointed out, however, there is no *a priori* reason to assume that the transphonologization of the tones be extended to other contexts, indicating that our findings may still be consistent with the tonogenetic account to the extent that the effect is specific to a particular prosodic context. With this possibility left open, in what follows, we discuss how some of our findings may lend further support to the prosodic account and take support away from the segmental-level transphonologization account.

First, a support of the prosodic account comes from the fact that the F0 difference due to the lenis–aspiration distinction is drastically reduced to the level of F0 perturbation in the non-prominent (unfocused) phrase-medial position. In the prosodic account, this innovation comes from an exclusive use of post-lexical tonal features for the stop distinction in prosodic contexts in which tones are assigned by the intonational phonology and therefore available to the speakers for phonological use. This means that the low-level F0 perturbation may occur in a prosodically weak context (unfocused and phrase-medial) in which post-lexical tones are neither specified by the intonational phonology nor transferred from the phrase-initial position. But the small F0 difference is difficult to explain in a system assumed in a tonogenetic account in which post-lexical tones have become primary phonetic cues for segmental contrasts across the board and are therefore available for phonetic implementation of F0 for phonological use in the segmental phonology. Furthermore, as we discussed above, the weak prosodic context is precisely where the segmental voicing feature (as reflected in VOT and %-voicing-in-closure) comes into play, showing a sufficient phonetic contrast along the voicing continuum across speakers of the different age and gender groups, which would complement the lack of available post-lexical tones for segmental distinction. This also implies that the segmental voicing feature (as implemented by VOT and %-voicing-in-closure) still plays a role in the phonological system, which is, if anything, more consistent with the prosodic account than with the transphonologization account in that the latter assumes no use of VOT for the lenis–aspirated stop distinction, though it does not specifically rule out its use in non-initial positions.

Second, the sort of focus-induced phonetic augmentation of F0 difference observed here does not directly reflect a phonological enhancement of the assumed targets of tonal features. In the introduction, we hypothesized that if tones have become primary phonetic features used for segmental contrasts, an L tone would become lower and an H tone higher in the prominent (focused) condition than in the non-prominent (unfocused) condition, showing a bidirectional polarization of tonal contrast. This prediction was based on the assumption that the focus-induced prominence regulates the phonetic realization of a distinctive feature in a direction to reach its assumed phonetic target in full [10, 11, 27, 29]. Such a bidirectional polarization effect was indeed found in Kyungsang (‘Gyeongsang’) Korean which employs tones as part of a lexical pitch accent system [29], and in Mandarin Chinese which employs lexical tones [31] (see also [59]). But the results of the present study show a unidirectional increase for both the L and the H tones, though the difference is phonetically enlarged under focus. This unidirectional enhancement of tonal contrast is similar to that of intonationally specified tonal contrasts observed in English and German ([32, 33]), thus lending support to the view of the prosodic account that tones used by speakers for the lenis–aspirated distinction are the ones post-lexically specified by the intonational phonology.

It is also interesting to note that the unidirectional F0 enhancement is largely consistent with an enhanced F0 rising effect of the onset consonant-induced pitch perturbations (also known as *CF0*) observed in various other languages [48, 60, 61]—i.e., the vowel is produced with a higher F0 following the release of voiceless obstruents than of voiced ones. In particular, a prominence (or focus)-related augmentation of high CF0 after the voiceless stop has been found in languages with a two-way laryngeal contrast in which the voiced consonant is (frequently) produced with prevoicing during closure such as Tokyo Japanese [48], French and Italian [60, 61]. showed comparable CF0 effects in Khmer, Thai and Vietnamese which all have a three-way stop contrast (voiced, voiceless unaspirated, voiceless aspirated), regardless of whether the language was non-tonal (Khmer) or tonal (Thai and Vietnamese), although the magnitude of *CF0* was larger in non-tonal Khmer than in the tonal languages. Crucially [60, 61], proposed that the CF0 effect occurs across languages and may serve as important phonetic correlates of the laryngeal contrast, not because languages share a phonological feature, but because the effects are driven by a phonetic process stemming from an active laryngeal gesture to inhibit phonation. Languages, as [60, 61] noted further, may then vary in its fine-phonetic detail often due to its interaction with language-specific constraints. Seoul Korean, however, differs from these languages because in Seoul Korean the onset-induced F0 perturbation in a phrase-initial position turned into post-lexical tonal contrasts of H and L, which is assigned to an AP-initial position by the intonational phonology of the language. Nevertheless, the fact that both the aspirated and the lenis stops in Seoul Korean are phonetically voiceless (with a positive VOT) implies that the F0 of the following vowel is phonetically implemented in much the same way as the CF0 effects are, sharing the same phonetic underpinnings. This is independent of whether the stop is produced with H or L that are post-lexically assigned as CF0 effects may still be found in tonal languages as reported in [61]. Note that reviewers asked how intonational tones (e.g., H and L) may be assigned especially in phrase-initial positions in which there is no clear phonetic feature (e.g, VOT) other than F0 that distinguishes between the lenis and the aspirated stop. This is in fact a question that should be addressed by the theory of the intentional phonology of Seoul Korean developed in [12, 34]. But we assume that the abstract phonological information about the segmental difference between the lenis and the aspirated stops is stored in the lexicon, and the information is available post-lexically for the intonational grammar of the language to use to determine the intonational tones.

## 5. Conclusion

The present results on the ongoing sound change in present-day Seoul Korean have shown that speakers of different age and gender groups utilize the segmental voicing feature (as implemented by VOT) and the tonal feature (as implemented by F0) differently for the lenis–aspirated stop contrast, and that the way they use these features varies as a function of prosodic factors of prominence and position. The results therefore suggest that the ongoing sound change is intricately related to the interaction between phonetics and prosody (the phonetics–prosody interface). The sort of sound change under *the prosodic account* we have proposed here is fundamentally different from the purported ‘tonogenetic’ view of sound change in which the F0 difference has been exaggerated and developed into distinctive tones in the segmental phonology of the language. Given that the tonogenesis has long been rooted in the intonational phonology of Seoul Korean, we suggest that the intonationally defined tones (L vs. H) provide salient post-lexical phonetic support for the lenis–aspirated segmental distinction in the phrase-initial position. The young speakers may then have chosen the low-cost option of using the perceptually salient post-lexical tones in place of VOT, presumably driven by the principle of effort minimization. Thus, in the prosodic account, the ongoing sound change is driven initially by a shift from the use of the segmental voicing feature to that of post-lexical tones to the extent that the tones are available in particular prosodic contexts (i.e., phrase-initial positions), and subsequently by the spread of the tonal use to the non-initial position provided that the position is licensed for attracting the tones—i.e., in the prominent (focused) condition. It should be noted, however, that the prosodic account based on the findings of the present study must not be taken to be conclusive, especially because the size of the production data is quite limited, obtained from only 32 speakers in a laboratory setting. It is hoped that the present study has made a step toward understanding the phonetic and phonological nature of the ongoing sound change in present-day Seoul Korean, and sparking further research from a perspective that considers the phonetics–prosody interface.

## Supporting information

S1 Appendix (DOCX)

S1 Data (UT8)
