# Supplementary material for: An apparent-time study of an ongoing sound change in Seoul Korean: A prosodic account
Source: PLoS One. 2020 Oct 22;15(10):e0240682. doi: 10.1371/journal.pone.0240682 (PMC7580931; doi:10.1371/journal.pone.0240682)
Supplement: S1 Appendix — (DOCX) [file pone.0240682.s001.docx]

S1 Appendix

Table 4. Adjusted weights of the contrasts of the factors for data of the IP-initial and IP-medial positions.

| Factor | Level | Data | |
| --- | --- | --- | --- |
|  |  | IP-initial position | IP-medial position |
| C-type | aspirated | 0.51 | 0.51 |
|  | lenis | -0.49 | -0.49 |
| Focus | focus | 0.47 | 0.51 |
|  | unfocus | -0.53 | -0.49 |
| Gender | female | 0.51 | 0.51 |
|  | male | -0.49 | -0.49 |
| Age | younger | 0.49 | 0.48 |
|  | older | -0.51 | -0.52 |
| TimePoint | 25% | 0.50 | 0.50 |
|  | midpoint | -0.50 | -0.50 |

Table 5. The specifications of the final models. Statistical analyses were carried out with R 3.5.1

| Dependent variable | Model formula |
| --- | --- |
| VOT (IP-initial) | VOT ~ Gender * Age * Focus * C.type + (1 + C.type * Focus \| Speaker) + (1 + Gender + Focus + Age \| Item) |
| F0 (IP-initial) | F0_st ~ Gender * Age * Focus * C.type * TimePoint + (1 + Focus + C.type + TimePoint \| Speaker) + (1 + Gender + Age + TimePoint \| Item) |
| %-voicing-in-closure  (IP-medial) | PercentageVoicing-in-Closure ~ Gender * Age * Focus * C.type + (1 + C.type * Focus \| Speaker) + (1 + Gender + Focus + Age \| Item) |
| VOT (IP-medial) | VOT ~ Gender * Age * Focus * C.type + (1 + C.type * Focus \| Speaker) + (1 + Gender + Age * Focus \| Item) |
| F0 (IP-medial) | F0_st ~ Gender * Age * Focus * C.type * TimePoint + (1 + C.type + Focus * TimePoint \| Speaker) + (1 \| Item) |

Table 6. Results from the linear mixed-effect models for VOT in the IP-initial and IP-medial positions, and for percentage of voicing duration in closure (%-voicing-in-closure) in the IP-medial positions.

|  | IP-initial |  |  | IP-medial |  |  |  |  |  |
| --- | --- | --- | --- | --- | --- | --- | --- | --- | --- |
|  | VOT |  |  | VOT |  |  | %-voicing-in-closure | | |
|  | Estimate | SE | *t* | Estimate | SE | *t* | Estimate | SE | *t* |
| (intercept) | 68.0 | 2.1 | 31.6*** | 42.6 | 1.7 | 24.8*** | 30.7 | 1.7 | 17.5*** |
| Gender | 0.5 | 4.1 | 0.1 | -0.8 | 3.1 | -0.3 | -2.4 | 3.5 | -0.7 |
| Age | 4.9 | 4.1 | 1.2 | 0.3 | 3.2 | 0.1 | -4.7 | 3.5 | -1.3 |
| Focus | 18.8 | 2.5 | 7.4*** | 52.0 | 3.2 | 16.1*** | -49.3 | 2.1 | -23.4*** |
| C-type | 12.7 | 2.2 | 5.7*** | 24.2 | 2.4 | 10.1*** | -39.5 | 2.1 | -18.9*** |
| Gender:Age | -2.1 | 8.1 | -0.3 | -4.0 | 6.3 | -0.6 | 4.4 | 6.9 | 0.6 |
| Gender:Focus | 5.7 | 4.3 | 1.3 | 4.7 | 5.8 | 0.8 | 6.0 | 4.1 | 1.5 |
| Age:Focus | 4.8 | 4.2 | 1.1 | 5.0 | 5.9 | 0.8 | -2.1 | 4.2 | -0.5 |
| Gender:C-type | -13.0 | 3.5 | -3.7*** | -14.3 | 3.9 | -3.7*** | -1.9 | 4.0 | -0.5 |
| Age:C-type | -18.7 | 3.4 | -5.4*** | -10.2 | 4.1 | -2.5* | -2.6 | 4.2 | -0.6 |
| Focus:C-type | -4.2 | 4.0 | -1.1 | -13.0 | 4.6 | -2.8** | 74.6 | 4.3 | 17.2*** |
| Gender:Age:Focus | -12.6 | 8.3 | -1.5 | -15.5 | 11.6 | -1.3 | 3.6 | 8.2 | 0.4 |
| Gender:Age:C-type | 11.1 | 6.7 | 1.7 | 13.0 | 7.7 | 1.7 | 15.2 | 8.1 | 1.9 |
| Gender:Focus:C-type | -3.4 | 5.7 | -0.6 | -12.1 | 7.1 | -1.7 | -4.6 | 8.4 | -0.5 |
| Age:Focus:C-type | -0.8 | 5.6 | -0.1 | -18.2 | 7.6 | -2.4* | 8.8 | 8.7 | 1.0 |
| Gender:Age:Focus:C-type | 19.2 | 10.7 | 1.8**^＋^** | 12.4 | 14.2 | 0.9 | -22.9 | 16.9 | -1.4 |
| **^＋^**p=0.08, * p<.05, ** p<.01, *** p<.001 | | | |  |  |  |  |  |  |

Table 7. Results from the linear mixed-effect models for F0 in the IP-initial and IP-medial positions.

|  | IP-initial |  |  | IP-medial |  |  |
| --- | --- | --- | --- | --- | --- | --- |
|  | Estimate | SE | *t* | Estimate | SE | *t* |
| (Intercept) | 9.0 | 0.4 | 24.7*** | 8.0 | 0.4 | 22.3*** |
| Gender | 11.2 | 0.7 | 15.5*** | 11.0 | 0.7 | 15.3*** |
| Age | 1.1 | 0.7 | 1.5 | 1.1 | 0.7 | 1.6 |
| Focus | 3.2 | 0.3 | 10.8*** | 4.0 | 0.3 | 14.7*** |
| C-type | 5.5 | 0.3 | 21.5*** | 3.8 | 0.2 | 22.8*** |
| TimePoint | 0.0 | 0.0 | 0.7 | 0.0 | 0.0 | 0.2 |
| Gender:Age | 0.0 | 1.4 | 0.0 | 0.7 | 1.4 | 0.5 |
| Gender:Focus | 0.5 | 0.6 | 0.9 | 1.1 | 0.5 | 2.0 |
| Age:Focus | -0.7 | 0.6 | -1.2 | -0.5 | 0.5 | -0.9 |
| Gender:C-type | 1.2 | 0.5 | 2.3* | 0.2 | 0.3 | 0.5 |
| Age:C-type | -0.3 | 0.5 | -0.7 | 0.2 | 0.3 | 0.6 |
| Focus:C-type | 3.5 | 0.2 | 15.9*** | 5.3 | 0.2 | 32.9*** |
| Gender:TimePoint | -0.1 | 0.1 | -1.3 | 0.0 | 0.1 | 0.1 |
| Age:TimePoint | 0.3 | 0.1 | 3.5*** | 0.3 | 0.1 | 3.0** |
| Focus:TimePoint | -0.5 | 0.1 | -6.1*** | -0.6 | 0.1 | -7.7*** |
| C-type:TimePoint | 0.1 | 0.1 | 1.4 | 0.2 | 0.1 | 2.6* |
| Gender:Age:Focus | -0.6 | 1.1 | -0.6 | -1.1 | 1.1 | -1.1 |
| Gender:Age:C-type | -1.6 | 0.9 | -1.7 | 0.1 | 0.6 | 0.2 |
| Gender:Focus:C-type | -0.4 | 0.2 | -1.9 | -0.3 | 0.2 | -2.1* |
| Age:Focus:C-type | 0.9 | 0.2 | 3.8*** | 0.0 | 0.2 | 0.1 |
| Gender:Age:TimePoint | 0.0 | 0.2 | -0.2 | 0.0 | 0.2 | -0.3 |
| Gender:Focus:TimePoint | 0.0 | 0.2 | 0.3 | 0.1 | 0.2 | 0.6 |
| Age:Focus:TimePoint | 0.3 | 0.2 | 1.6 | 0.3 | 0.2 | 1.9 |
| Gender:C-type:TimePoint | -0.1 | 0.2 | -0.6 | -0.3 | 0.2 | -1.7 |
| Age:C-type:TimePoint | -0.6 | 0.2 | -3.6*** | -0.3 | 0.2 | -1.8 |
| Focus:C-type:TimePoint | 0.2 | 0.2 | 1.4 | 0.1 | 0.2 | 0.4 |
| Gender:Age:Focus:C-type | 0.6 | 0.3 | 1.8 | -0.5 | 0.3 | -1.4 |
| Gender:Age:Focus:TimePoint | -0.1 | 0.3 | -0.2 | 0.2 | 0.3 | 0.5 |
| Gender:Age:C-type:TimePoint | 0.3 | 0.3 | 0.8 | 0.3 | 0.3 | 0.8 |
| Gender:Focus:C-type:TimePoint | 0.1 | 0.3 | 0.2 | -0.3 | 0.3 | -1.1 |
| Age:Focus:C-type:TimePoint | -0.5 | 0.3 | -1.5 | -1.0 | 0.3 | -3.0** |
| Gender:Age:Focus:C-type:TimePoint | 0.5 | 0.7 | 0.7 | 0.3 | 0.6 | 0.5 |
| * p<.05, ** p<.01, *** p<.001 |  |  |  |  |  |  |
